# HPV Oncogene Manipulation Using Nonvirally Delivered CRISPR/Cas9 or *Natronobacterium gregoryi* Argonaute

**DOI:** 10.1002/advs.201700540

**Published:** 2018-05-18

**Authors:** Yeh‐Hsing Lao, Mingqiang Li, Madeleine A. Gao, Dan Shao, Chun‐Wei Chi, Dantong Huang, Syandan Chakraborty, Tzu‐Chieh Ho, Weiqian Jiang, Hong‐Xia Wang, Sihong Wang, Kam W. Leong

**Affiliations:** ^1^ Department of Biomedical Engineering Columbia University New York NY 10027 USA; ^2^ Department of Biomedical Engineering CUNY—City College of New York New York NY 10031 USA; ^3^ Department of Systems Biology Columbia University Medical Center New York NY 10032 USA

**Keywords:** CRISPR/Cas9, gene delivery, gene editing, gene silencing, human papillomavirus (HPV), *Natronobacterium gregoryi* Argonaute (NgAgo)

## Abstract

CRISPR/Cas9 technology enables targeted gene editing; yet, the efficiency and specificity remain unsatisfactory, particularly for the nonvirally delivered, plasmid‐based CRISPR/Cas9 system. To tackle this, a self‐assembled micelle is developed and evaluated for human papillomavirus (HPV) E7 oncogene disruption. The optimized micelle enables effective delivery of Cas9 plasmid with a transient transgene expression profile, benefiting the specificity of Cas9 recognition. Furthermore, the feasibility of using the micelle is explored for another nucleic acid‐guided nuclease system, *Natronobacterium gregoryi* Argonaute (NgAgo). Both systems are tested in vitro and in vivo to evaluate their therapeutic potential. Cas9‐mediated E7 knockout leads to significant inhibition of HPV‐induced cancerous activity both in vitro and in vivo, while NgAgo does not show significant E7 inhibition on the xenograft mouse model. Collectively, this micelle represents an efficient delivery system for nonviral gene editing, adding to the armamentarium of gene editing tools to advance safe and effective precision medicine‐based therapeutics.

## 1 Introduction

Gene mutations and allele variations usually contribute to disease heterogeneity, which may result in treatment failure; thus, precision medicine‐based therapeutic approaches have increasingly attracted attention, especially since the CRISPR/Cas9 system was reported.^1^ The CRISPR/Cas9 system allows targeted genome editing, including frameshift knockout, gene insertion, and alteration, under the guidance of a specific guide RNA (gRNA).^1^ Since the first system engineered from *Streptococcus pyogenes* Cas9 endonuclease,^2^ many Cas9 variants (e.g., *Staphylococcus aureus* Cas9^3^), analogues (e.g., CRISPR/Cpf1^4^ and FEN‐1/FokI fused endonuclease^5^) have emerged. To date, delivery of these gene editing systems relies mainly on viral vectors or electroporation.^6^ While being efficient, these methods hold drawbacks that may hinder clinical translation. Viral transduction may introduce random insertions as well as immunogenicity and toxicity,^7^ while electroporation may cause high cell death rates and is not applicable for systemic delivery.^8^ Nonviral delivery offers an alternative. Yet, nonviral delivery of these systems remains a challenge, especially for the plasmid‐based CRISPR/Cas9 system. A recently published work reports that the commercially available liposomal carrier shows limited efficiencies in several cell lines with the Cas9 plasmid.^9^ In addition, this widely used carrier demonstrates significantly low gene‐targeting specificity with the plasmid‐based CRISPR/Cas9 system, which is 1.6‐fold to 20‐fold lower than the specificity with mRNA‐based and protein‐based systems. Therefore, although more studies on nonviral delivery of the CRISPR/Cas9 system have been published recently, they were mostly based on mRNA‐based and protein‐based systems and did not address the aforementioned issues on Cas9 plasmid delivery.^10^

**Figure 1:**
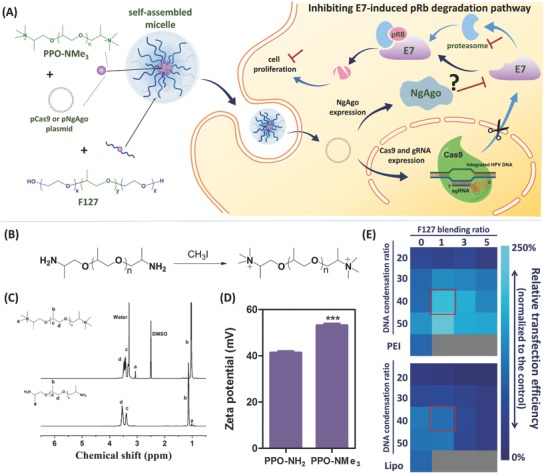
Design and optimization of the proposed micellar system for gene manipulation. A) HPV oncogene manipulation with the micelle proposed in this study. B) Synthesis, C) ^1^H NMR, and D) zeta potential characterization of PPO‐NMe_3_. Data are represented as average ± standard error of mean (SEM; *n* = 3). Two‐tailed Student's *t*‐test was used for *p*‐value calculation. The significant level is represented as ∗∗∗ (*p* < 0.001). E) Influence of DNA condensation and F127 blending ratios on micelle's Cas9 transfection efficiency.

Conventional thinking of a nonviral carrier design for plasmid delivery is to have a polycation with a high charge density, so that the carrier can prevent the plasmid from degradation in order to achieve higher transgene expression.^11^ However, this may have a negative effect on plasmid‐based CRISPR/Cas9 delivery because of the relatively large size of Cas9 plasmid. Moreover, a sustained Cas9 expression could also lead to undesired off‐targeting.^9^ Herein, hypothesizing that a carrier with a lower charge density may be a better choice for Cas9 plasmid delivery, we designed a self‐assembled micelle, composed of quaternary ammonium‐terminated poly(propylene oxide) (PPO‐NMe_3_) and amphiphilic Pluronic F127, optimized for delivering the plasmid‐based gene editing and manipulation systems (**Figure** **1**A). The composition of the micelle was optimized and tested on a human papillomavirus (HPV) model to target HPV18‐E7 oncogene. HPV E7 is a well‐known oncoprotein that inhibits retinoblastoma protein (Rb) via the ubiquitin‐proteasome pathway and leads to aberrant cell proliferation.^12^ In conjunction with an all‐in‐one Cas9 construct (termed pCas9), encoding Cas9‐green fluorescent protein (GFP) and gRNA against HPV18‐E7, the optimized micelle efficiently disrupted the E7 oncogene in HeLa cell's genome, thereby inhibiting the downstream cancerous activity both in vitro and in vivo. Furthermore, we evaluated the delivery potential of our micellar carrier with *Natronobacterium gregoryi* Argonaute (NgAgo). NgAgo was first reported as a gene editing enzyme.^13^ We initially sought to benchmark these two different gene editing systems using the same, optimized nonviral carrier. Unfortunately, we observed the same irreproducibility of the NgAgo system in gene editing as reported by several other groups.^14^ Learning that NgAgo may interfere with RNA rather than double‐stranded DNA (dsDNA) in a cell‐free assay,^15^ we subsequently evaluated the gene silencing potential of the NgAgo system on the same HPV model with the F127/PPO‐NMe_3_ micelle.

## 2 Results

### 2.1 Micellar Carrier Design and Optimization

To condense the plasmids for gene manipulation, we chose a linear, low charge density PPO as it matches the hydrophobic part of Pluronic family. We first introduced quaternary ammonium to the terminus of PPO‐NH_2_, as quaternary ammonium possesses stronger nucleic acid‐binding affinity^16^ without increasing the charge density (Figure 1B). The modified product, quaternary ammonium‐terminated PPO (PPO‐NMe_3_), was confirmed with ^1^H NMR (Figure 1C; the peak b of PPO‐NMe_3_ shifted downfield because of the solvent difference between PPO‐NH_2_ and PPO‐NMe_3_ samples; PPO‐NH_2_ was dissolved in CDCl_3_, while PPO‐NMe_3_ was dissolved in dimethyl sulfoxide‐*d*_6_ (DMSO‐*d*_6_) along with a more positive zeta potential (Figure 1D). To boost transfection efficiency and enhance the stability, we added the FDA‐approved F127 because of the similar molecular weights between its hydrophobic PPO segment and the PPO‐NMe_3_ (*M*_n_ = 4000), as well as its biocompatibility and extensive application in medicine, particularly drug delivery in vivo.^17^

The mixing of PPO‐NMe_3_, F127, and the plasmid produced micelles formulated through the hydrophobic and electrostatic self‐assembly of the three components. The DNA condensation ratio (PPO‐NMe_3_/plasmid) and the F127 blending ratio (F127/PPO‐NMe_3_) were important factors determining the transfection efficiency. We systematically screened these two factors and checked their influence on the micelles' size, polydispersity (PDI), and zeta potential. We compared the transfection efficiency of Cas9‐GFP plasmid (w/o gRNA) in HPV18^+^ HeLa cells with that of branched polyethylenimine (PEI; 25 kD) and Lipofectamine 2000.

When the DNA condensation ratio was at 10 (w/w), the complex without F127 was smaller than 200 nm in reduced serum medium (Opti‐MEM) because of its negatively charged surface (Figure S1, Supporting Information), but this yielded a limited Cas9 transfection efficiency (Figure S2A, Supporting Information). By contrast, when the DNA condensation ratio was over 20, PPO‐NMe_3_/pCas9 became a positively charged complex, and F127 conferred colloidal stability to the nanocomplex (Figure S1, Supporting Information). This can be explained by the polyethylene glycol (PEG) corona that imparts colloidal stability and reduces nonspecific protein adsorption.^18^

Increasing the F127 blending ratio did not significantly reduce the size of the complex or change its zeta potential. However, reduced transfection efficiency was observed (Figure 1E), likely attributed to the undesired blocking effect of the PEG corona on endocytosis.^19^ When the F127 blending ratio was kept at 1 while the DNA condensation ratio was increased to over 30, the micelle was able to transfect HeLa cells with an efficiency of 101.7 to 238.3%, and 60.9 to 121.7%, compared with the optimized formulation of PEI and Lipofectamine 2000, respectively (Figure 1E; Figure S2A, Supporting Information). Whereas the transfection efficiency of the micelle plateaued at the DNA condensation ratio of 50, its cytotoxicity also increased. As a result, we chose the ratios of 40 for DNA condensation and 1 for F127 blending for the subsequent experiments. Under this condition, the micelle was able to transfect 30.3 ± 2.93% of the HeLa cells with pCas9‐GFP (24 h post‐transfection; data obtained from three independent experiments), and was less toxic than PEI, with 16.5 ± 4.21% versus 72.7 ± 0.28% cell death at the same polymer dose ([cationic polymer] = 40 µg mL^−1^; Figure S2B, Supporting Information). The micelle was stable in Opti‐MEM as well as in the complete medium with 10% fetal bovine serum (FBS) (Figure S2C,D, Supporting Information), and could prevent the Cas9 plasmid from enzymatic degradation under the physiological DNase I condition (Figure S3, Supporting Information).

### 2.2 Cas9 Delivery Using F127/PPO‐NMe_3_/pCas9 Micelle

The cellular uptake and protein expression profiles of the optimized micellar formulation (F127/PPO‐NMe_3_/pCas9 = 40/40/1, w/w/w) were then examined to characterize the nanocomplex internalization and Cas9 turnover rate. To study the cellular uptake, a similar Cas9 construct without a GFP tag (Addgene #62934) was used. We first stained it with TOTO3 using our previously established DNA staining protocol^20^ and formed the micelle with fluorescein isothiocyanate (FITC)‐labeled F127. After 4 h incubation at 37 °C, the HeLa cells efficiently internalized the micelles, and the TOTO3‐labeled plasmid could be observed in both the cytoplasm and nucleus (Figure S4A, Supporting Information). Furthermore, the uptake was quantitated using fluorescence‐activated cell sorting (FACS). While naked Cas9 plasmid showed negligible endocytosis, the micelle delivered the Cas9 plasmid to 91.0 ± 6.61% of the cells after 4 h incubation (Figure S4B, Supporting Information).

**Figure 2:**
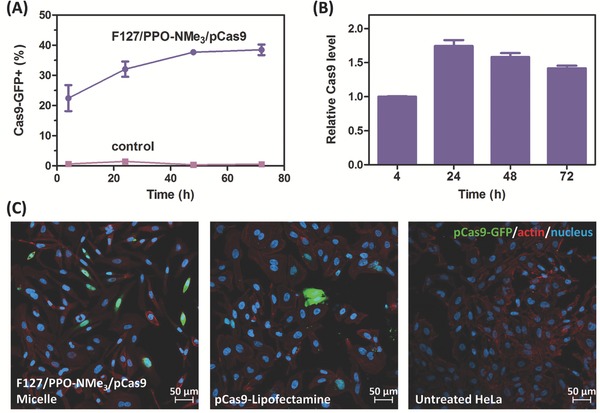
Cas9 transfection using optimized F127/PPO‐NMe_3_/pCas9 (40/40/1) micelle. A) Transgene expression kinetics in HeLa cells. B) Relative Cas9 expression level in transfected HeLa cells (data normalized to the Cas9‐GFP fluorescent intensity at 4 h post‐transfection). Data are represented as average ± SEM (*n* = 3). C) CLSM images of HeLa cells incubated with F127/PPO‐NMe_3_/pCas9 micelle or pCas9‐loaded Lipofectamine for 24 h.

Next, we measured the Cas9‐GFP protein expression profile using FACS. As shown in **Figure** **2**A, 22.5 ± 3.05% of HeLa cells started expressing Cas9‐GFP at 4 h post‐transfection. To quantify the Cas9 expression level, we normalized the mean fluorescent intensity of the GFP^+^ cells at each time point to that at 4 h. The Cas9 expression peaked at 24 h with 32.1% transfection efficiency (Figure 2A,B). Unlike our observation, previous work has shown that Cas9 was cumulatively expressed in HEK293T cells when using the plasmid‐loaded liposomal carrier.^9^ This suggests that this micellar approach may have lower off‐target effects on CRISPR/Cas9 editing due to its faster turnover rate. We also measured the protein colocalization by confocal microscopy. Similar to the cellular uptake result, Cas9‐GFP protein was found in the cytoplasm and also colocalized in the nucleus because of the nuclear localization signal (NLS) (Figure 2C). Furthermore, compared with Lipofectamine‐treated cells, more micelle‐treated cells were Cas9‐GFP positive at 24 h post‐transfection, showing the micelle's superior transfection efficiency (Figure 2C).

### 2.3 HPV Oncogene Disruption Using F127/PPO‐NMe_3_/pCas9 Micelle

**Figure 3:**
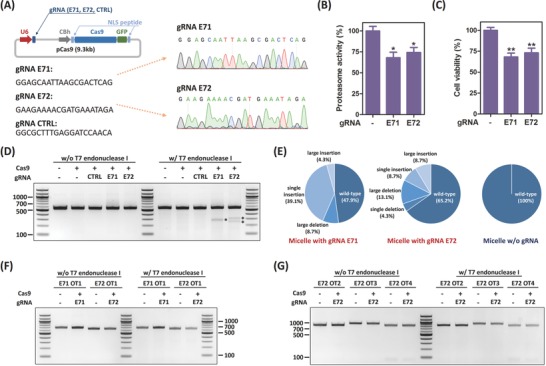
HPV E7 disruption using F127/PPO‐NMe_3_/pCas9 micelle. A) pCas9 construct design and gRNAs. B) Proteasome activity of Cas9‐transfected HeLa cells. Data are represented as average ± SEM (*n* = 3). C) Cell viability of Cas9‐transfected HeLa cells. Proteasome activity and cell viability were measured at 72 h post‐transfection. Data are represented as average ± SEM (*n* = 4). One‐way ANOVA with Dunnett's multiple comparison test was used for *p*‐value calculation. The significant level is represented as ∗ (*p* < 0.05); ∗∗ (*p* < 0.01). D) T7EI assay to verify E7 gene disruption. E) Sequencing analysis of micelle‐transfected cells. T7EI assays to verify the off‐targeting based on the prediction using F) Cas9‐OFFinder and G) BLAST (for (C) through (G), the cells were sorted using GFP marker at 24 h post‐transfection and genomic DNAs were extracted at 96 h post‐transfection).

To target HPV18‐E7 oncogene, we built the all‐in‐one pCas9 construct, encoding both Cas9‐GFP gene and a specific gRNA. Briefly, we followed the method published by Ran et al.,^21^ and used their pSpCas9(BB)‐2A‐GFP plasmid to clone three different types of gRNAs: two targeting HPV18‐E7 oncogene (gRNAs E71 and E72) and one control (**Figure** **3**A). Although a nucleotide mismatch was designed at the 5′‐end of gRNA E71's targeting region, due to gRNA E71's need for transcription with the human U6 promoter, it should not affect the Cas9 recognition ability as previously reported.^22^ The cloned sequences of these gRNAs, as well as the plasmid backbone, were verified by Sanger sequencing technique (Figure 3A; Figure S5, Supporting Information).

To evaluate the potential of our micelle for therapeutic applications, we chose to target HPV18 E7 oncogene as a therapeutic model. HPV E7 is known to promote aberrant cell proliferation and therefore HPV pathogenesis.^12^ In cancerous transformations, E7 protein directly binds to Rb tumor suppressor, resulting in Rb degradation. Hypothesizing that disruption of the E7 oncogene salvages Rb expression and hence inhibits cancer cell proliferation, we designed two gRNAs targeting the E7 locus. In HPV‐infected cells, HPV E7 regulates Rb protein via the ubiquitin‐proteasome pathway. Ubiquitination directly activates proteasome 26S and induces protein degradation.^23^ Additionally, E7 boosts the proteasome's enzymatic activity through interaction with the S4 ATPase subunit domain on proteasome 26S.^24^ Thus, we first tested if our nonviral approach on E7 oncogene disruption could inhibit the proteasome activity. At 72 h post‐transfection, we found the proteasome activity reduced by 31.9% and 25.7% on the cells treated with gRNAs E71 and E72, respectively (Figure 3B). As Rb is a tumor suppressor, rescuing Rb expression levels could result in inhibition of cell proliferation. As presented in Figure 3C, both the groups treated with gRNAs E71 and E72 grew significantly slower, reaching 68.1% and 73.1% cell viability of the Cas9 control.

We next investigated the mutation induced by our F127/PPO‐NMe_3_/pCas9 micelle. Following a published protocol,^22^ we sorted the live, GFP^+^ HeLa cells and then reseeded the cells on a 96‐well plate. We also introduced a gRNA control to prevent possible false‐positive results in the following experiments. The genomic DNA of each sample was extracted at 96 h post‐transfection. After amplification and purification, the polymerase chain reaction (PCR) products were reannealed, and mutations were detected with T7 endonuclease I. The expected PCR product was 551 bp (Table S1, Supporting Information); after T7 endonuclease I digestion, the expected Cas9‐induced cleaved products of the E71 and E72 groups were 281 + 270 bp and 303 + 248 bp, respectively. As shown in Figure 3D, mutations were indeed detected in the cells treated with Cas9 and E7‐targeting gRNAs, but not in either group with Cas9 only or Cas9 with the control gRNA. In addition, sequencing identified the mutation; 23 clones from the cells treated with Cas9 and E7‐targeting gRNAs were analyzed. Among these, we observed 52.1% (12/23) and 34.8% (8/23) of the clones mutated in the E71 and E72 groups, respectively. Sequencing readings across the predicted double‐strand break site on the E71 group showed 2 clones (8.7%) with large deletions, 9 (39.1%) with single‐base insertions, 1 (4.3%) with large insertion, and 11 (47.9%) wild‐type clones (Figure 3E; Figure S6A, Supporting Information). Similarly, for the E72 group, sequencing reads contained 1 site with single‐base deletion, 3 (13.1%) with large deletions, 2 with single‐base insertions, 2 with large insertions, and 15 (65.2%) wild‐type clones (Figure 3E; Figure S6B, Supporting Information). We also sequenced the cells treated only with Cas9 and found that all were wild‐type in the 10 identified clones (Figure 3E; Figure S6C, Supporting Information). These results confirmed again that our micelles could deliver pCas9 and precisely disrupt the HPV E7 oncogene. Furthermore, as Lipofectamine was more potent than PEI, we compared our approach with Lipofectamine side‐by‐side on T7EI assay and sequencing. On T7EI assay, we detected these products on both micelle‐transfected and Lipofectamine‐transfected HeLa cells with a comparable efficiency (Figure S7A, Supporting Information). Nevertheless, sequencing data showed that the proposed micelles induced more disruptions than the Lipofectamine‐induction on the transfected cells (34.8% vs 26.1%; Figure S7B, Supporting Information).

On the other hand, as aforementioned, the Cas9 expression profile on the F127/PPO‐NMe_3_/pCas9 micelle is more likely to be transient due to a faster turnover rate (Figure 2B). We hypothesized that our micellar carrier might therefore have a reduced off‐target effect and verified the possible off‐target sites on both gRNAs E71 and E72. Using Cas‐OFFinder, an off‐target prediction website,^25^ we also identified potential off‐target sites. With a parameter tolerant to 2‐bp mismatch, one potential off‐target site was identified on each gRNA (Figure S8A, Supporting Information). However, as shown in Figure 3F, no significant editing occurred in these two groups. Moreover, we tested different potential off‐target sites directly identified by blasting our gRNA's 20‐mer‐targeting region with the protospacer adjacent motif (PAM). Using the default setting on the NCBI BLAST website as the criteria, more potential off‐target sites were identified on gRNA E72, rather than gRNA E71. Since gRNA E72 had an increased possibility of recognizing a wrong position, we further evaluated the off‐targeting on its top 3‐ranked predicted sites on chromosomes 2, 6, and 10 (Figure S8B, Supporting Information). Again, no detectable editing was observed on these three sites (Figure 3G).

### 2.4 HPV E7 mRNA Knockdown Using F127/PPO‐NMe_3_/pNgAgo Micelle

Although CRISPR/Cas9 system is a powerful tool for genome editing, the size and GC‐rich sequence preference of Cas9 endonuclease limits its efficacy and target selection.^6^ More studies on improving the fidelity and finding an alternative have been reported.^1^, ^3^, ^4^ To broaden the applicability of our delivery system, we explored other gene editing alternatives. NgAgo was originally reported as one alternative that could achieve higher fidelity because of no target sequence preference.^13^ However, like other groups reported previously,^14^ we failed in demonstrating gene editing with NgAgo, even using the virally transduced NgAgo with NLS signaling peptides (FLAG–NgAgo–NLS) on our HeLa model. With the two NgAgo‐preferred DNA guide strands (gDNAs), modified from gRNAs E71 and E72, there was no detectable gene mutation caused by FLAG–NgAgo–NLS on T7EI assay (Figure S9, Supporting Information; gDNAs' sequence listed in Table S1, Supporting Information).

In trying to understand the reasons for the failure of NgAgo in gene editing, we checked the NgAgo protein sequence using Protein BLAST and found a PIWI domain on this protein, implying that it may have a function similar to RNase H.^26^ To prove this, again with the virally transduced NgAgo–NLS, we found a reduction on E7 mRNA expression level with the two gDNAs (Figure S10, Supporting Information). This is in agreement with the recently reported result demonstrating that NgAgo as a DNA‐guided ribonuclease.^15^ As an RNA effector, NgAgo with a NLS motif might reduce its efficacy. Thus, we built a nonviral NgAgo–enhanced GFP (EGFP) construct without NLS (pNgAgo–EGFP) for gene knockdown applications (Figure S11, Supporting Information). This construct could be condensed and formed a micelle when incubated with PPO‐NMe_3_ and F127. With the previously optimized condition (F127/PPO‐NMe_3_/plasmid = 40/40/1), pNgAgo–EGFP‐encapsulated micelle held the size and zeta‐potential similar to those of the micelle carrying pCas9 (Figure S12A, Supporting Information). We also observed similar shape and size between the micelles carrying pNgAgo–EGFP and pCas9 under transmission electron microscopy measurement (Figure S12B, Supporting Information). Furthermore, the encapsulation of pNgAgo–EGFP did not affect the micelle's cellular uptake kinetics, and the micelle carrying pNgAgo–EGFP could be internalized by the cell efficiently during the transfection process (Figure S13, Supporting Information).

**Figure 4:**
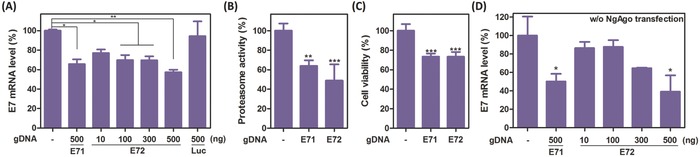
HPV E7 knockdown using the optimized micelle with the NgAgo system. A) HPV18‐E7 mRNA expression level in NgAgo‐transfected HeLa cell at 48 h post‐gDNA transfection. Data are presented as average ± SEM (*n* = 3). B) Proteasome activity of NgAgo‐transfected HeLa cells. Data are presented as average ± SEM (*n* = 4). C) Cell viability of NgAgo‐transfected HeLa cells. Data are presented as average ± SEM (*n* = 6). Proteasome activity and cell viability were measured at 72 h post‐NgAgo transfection. D) HPV18‐E7 mRNA expression level in the cells transfected with gDNA only (*n* = 4). One‐way ANOVA with Dunnett's multiple comparison test was used for p‐value calculation. The significant level is represented as ∗ (*p* < 0.05); ∗∗ (*p* < 0.01); ∗∗∗ (*p* < 0.001).

For validation, we first transfected the HeLa cell using the optimized micelle with pNgAgo–EGFP. At 24 h post‐transfection, the gDNAs were then introduced using the same optimized micelle. After culturing for another 48 h, total mRNAs were extracted and DNase I‐treated for reverse‐transcription quantitative PCR (RT‐qPCR) quantitation. As shown in **Figure** **4**A, the HPV18‐E7 mRNA expression levels in the cells treated with gDNAs E71 and E72 were reduced by 34.3 ± 4.93% and 42.7 ± 2.62% compared with the control without gRNA transfection, respectively. When treated with a gDNA control (Luciferase‐targeting; sequence listed in Table S1, Supporting Information), the HPV18‐E7 mRNA expression was not significantly inhibited. In addition, NgAgo‐mediated RNA interference was gDNA‐dose dependent. Even at a low dosage of 10 ng with the potent gDNA (E72), our micellar platform still inhibited HPV18‐E7 expression by 22.9 ± 3.63% (Figure 4A). To avoid false‐positive results, we also validated the RT‐qPCR quantitation with another primer pair published previously,^27^ and tested the mRNA inhibition using Lipofectamine 2000. With another primer pair, we obtained similar results with less than 3% variations (2.7% for the E71 group, 0.9% for the E72 group, and 0.1% for the Luc control; Figure S14, Supporting Information). Lipofectamine also gave a similar, but less efficient, result for NgAgo‐mediated RNA inhibition (Figure S15, Supporting Information), indicating that our micellar platform is more effective for both Cas9 and NgAgo applications. Similar to Cas9‐mediated knockout, knockdown of HPV18‐E7 oncogene inhibited both proteasome and cell proliferation as well. Nonvirally delivered NgAgo reduced the proteasome activity by 36.1 ± 2.89% and 51.2 ± 8.37% with the gDNAs E71 and E72, respectively (Figure 4B). A similar result was observed for cell proliferation inhibition; the cells treated with both gDNAs showed lower cell viability at 72 h post‐NgAgo transfection (73.5% and 73.3%; Figure 4C). However, in the absence of NgAgo transfection, micellar delivery of gDNA alone could mediate gene knockdown at the highest dosage of 500 ng (Figure 4D), although this phenomenon was not observed in the other study using a Zebrafish model.^[14c]^ We must allow for the possibility that the gDNA acts like an antisense oligonucleotide to interfere with the mRNA level.

### 2.5 Tumor Suppression Using Micelle‐Delivered Cas9 or NgAgo

**Figure 5:**
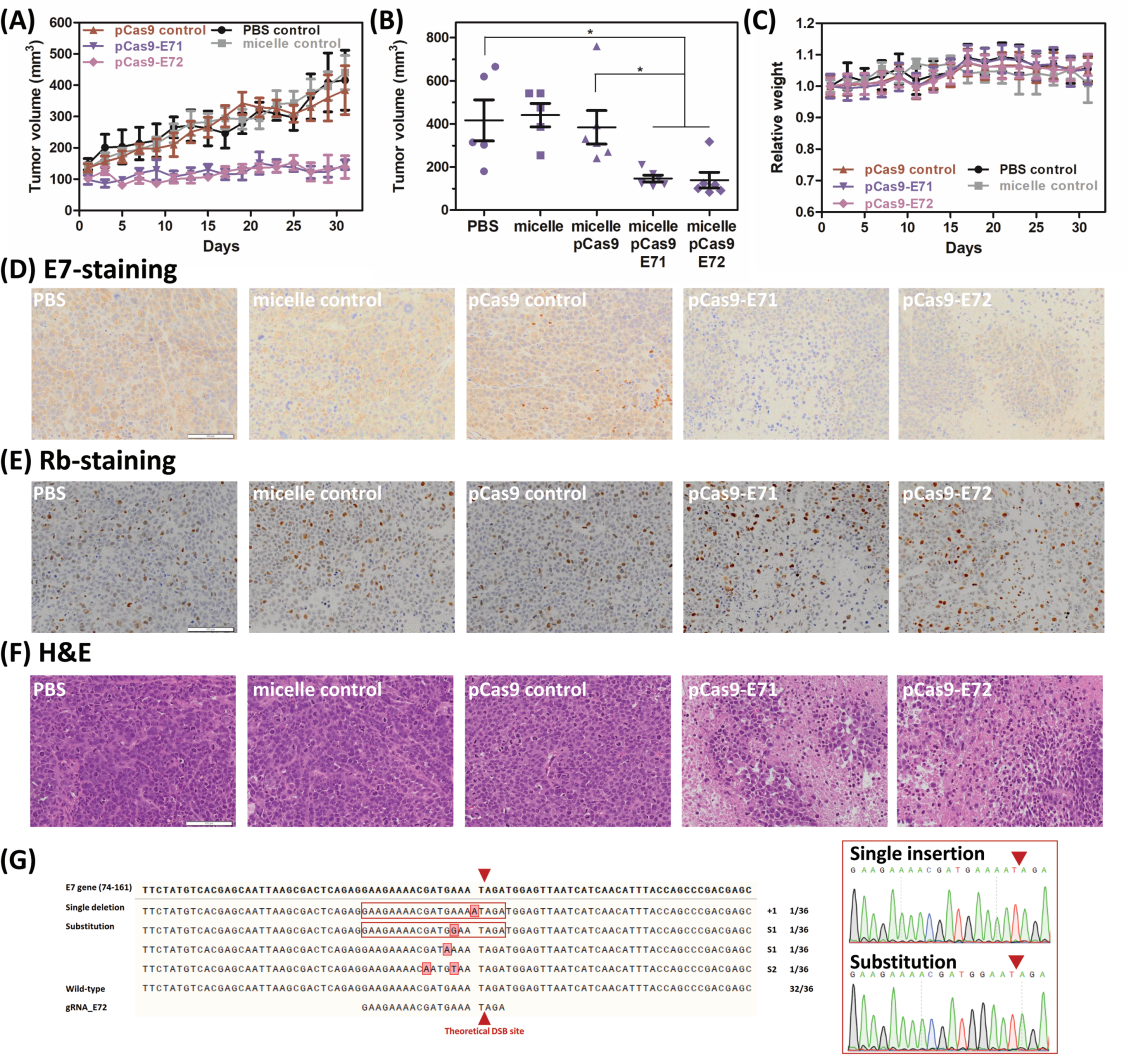
Therapeutic effect of using micelle‐delivered Cas9 on the HeLa xenograft model. A) Tumor growth in response to locally administered micelle, pCas9‐loaded micelle, and E7‐targeting pCas9‐loaded micelles (E71 and E72). B) Tumor volume comparison on the day when mice were sacrificed (day 31). C) Changes in body weight throughout the whole treatment course. Data are presented as average ± SEM (*n* = 5 for PBS and micelle controls; *n* = 6 for pCas9 control and the E71, E72 groups). One‐way ANOVA with Dunnett's multiple comparison test was used for *p*‐value calculation. The significant level is represented as ∗ (*p* < 0.05). Representative images of D) E7‐stained, E) Rb‐stained, and F) H&E‐stained tumor tissue sections. Scale bar represents 100 µm. G) Sequencing analysis for the genomic DNAs extracted from the tumor tissue treated with micelle‐delivered pCas9 and gRNA E72 (insets: representative sequencing results).

After observing micelle‐delivered Cas9 and NgAgo inhibition of the HPV18‐E7 expression in vitro, we further examined these systems in vivo. First, we tested the Cas9 system on a subcutaneous HeLa xenograft model. The E7‐targeting pCas9‐loaded micelle (equivalent plasmid dosage = 5 µg per mouse) was administered intratumorally every four days for a month, in comparison with blank micelle and micelle carrying control Cas9 construct (w/o gRNA). As shown in **Figure** **5**A, micelle‐delivered Cas9 with E7‐targeting gRNA efficiently delayed the tumor growth, while the group treated with either the carrier or the Cas9 control did not show any significant tumor inhibition (*p* < 0.05; One‐way ANOVA). By day 31, the tumors in the E71 and E72 groups only reached 38.2 ± 3.99 and 36.2 ± 9.42% of the tumor volume of the Cas9 control group (Figure 5B). Additionally, we did not observe significant weight loss and organ toxicity on the treated mice (Figure 5C; Figure S16, Supporting Information). Histological results correlated with the result of tumor growth measurement. The tumor tissues extracted from the control groups showed strong E7 expression, whereas tumors from mice treated with E7‐targeting pCas9 (gRNA E71 and E72) held reduced E7 expression (Figure 5D). Furthermore, compared with the controls, we also observed the Rb restoration and increased coagulative necrosis in both E71 and E72 groups (Figure 5E,F). In addition to qualitative histological measurements, we also sequenced the tumor tissues extracted from E72 and Cas9 control groups. For the E72 group, we detected four Cas9‐induced mutations from randomly selected 36 colonies (Figure 5G). By contrast, all of the selected colonies from the tumor treated with Cas9 control were wild‐type (Figure S17, Supporting Information). Notably, we detected an A to G substitutional mutation at the target 10 nucleotide upstream of the PAM motif in E72 group and the controls from both in vitro and in vivo experiments (Figures S6B,C and S17, Supporting Information), which may be due to the heterogenicity of HeLa itself, so we excluded those from the calculation.

**Figure 6:**
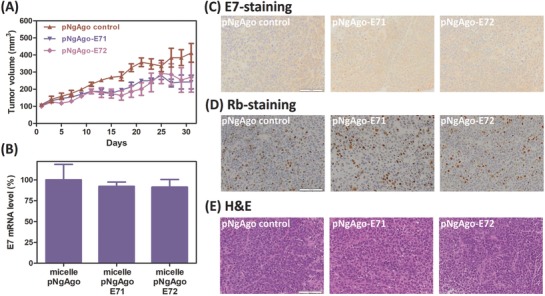
Therapeutic effect of using micelle‐delivered NgAgo on the HeLa xenograft model. A) Tumor growth in response to locally administered pNgAgo‐loaded micelle and pNgAgo‐loaded micelles with E7‐targeting gDNAs (E71 and E72). B) HPV18‐E7 mRNA expression level in the NgAgo‐treated tumor data are presented as average ± SEM (*n* = 5). One‐way ANOVA with Dunnett's multiple comparison test was used for *p*‐value calculation. Representative images of C) E7‐stained, D) Rb‐stained, and E) H&E‐stained tumor tissue sections. Scale bar represents 100 µm.

We next tested if the treatment of micelle‐delivered NgAgo could also give similar therapeutic outcome. The micelle carrying NgAgo or the corresponding gDNA was administered locally in a similar fashion. The treatment course included two injections, and each course was given every four days for a month as well. The pNgAgo‐loaded micelle was first injected, and gDNA‐loaded micelle given two days later. Each injection contained equivalent amounts of 5 µg DNA for each mouse. Similar to Cas9 treatment, no significant changes on body weight and organ toxicity were observed for NgAgo treatment (Figure S18A,B, Supporting Information). However, from a month‐long measurement, although micelle‐delivered NgAgo with gDNA showed slight tumor inhibition, the difference between groups was not significant (**Figure** **6**A; Figure S18C, Supporting Information). Also, only <10% E7 inhibition was observed in the group treated with both pNgAgo and E7‐targeting gDNA (7.8% and 8.7% for the E71 and E72 groups, respectively; Figure 6B). We also did not detect any notable E7 reduction nor Rb restoration from the histological analyses (Figure 6C,D). Moreover, when compared with the control, no obvious necrosis was found in the tumors treated with both NgAgo and E7‐targeting gDNA (Figure 6E).

## 3 Discussion

Although nonviral gene delivery is considerably mature for certain applications after almost three decades of development, it still encounters difficulty on efficiency and gene‐targeting specificity for the delivery of genome editing systems. Especially for the plasmid‐based CRISPR/Cas9 system, for example with the commonly used liposomal transfection reagent, a previously reported work has shown limited efficiencies (<15%) in the easy‐to‐transfect cell lines, such as U2OS and A549, and relatively low gene‐targeting specificity (off/on target ratio is ≈0.6–1.5).^9^ In this work, based on the hypothesis that a low charge‐density polycation could transfect those systems better with less off‐targeting, we have designed a self‐assembled micelle, containing quaternary ammonium‐modified PPO (PPO‐NMe_3_) and amphiphile F127 (Figure 1A). The quaternary ammonium modification (Figure 1B–D) benefits the DNA binding as well as enhances the cell‐penetration capability, without any undesirable increase of charge density. On the other hand, the Pluronic family has been widely used both in vitro and in vivo to transfect genes, to enhance the transfection efficiency of polycations, or to stabilize DNA/polycation complexes.^28^ Similarly here, F127 stabilizes the DNA/PPO‐NMe_3_ complex in the serum‐containing condition and improves the transfection efficiency. For the in vitro HPV model with pCas9, this approach holds superior transfection efficiency compared with both PEI and Lipofectamine 2000 (Figure 1E).

In addition to the efficiency, our micellar platform enables a faster protein turnover rate (Figure 2). The transient Cas9 expression does not affect the efficacy; the micelle‐delivered Cas9 is able to inhibit E7 oncoprotein's downstream proteasome pathway and thus slows down cervical cell proliferation (Figure 3B,C). Both T7EI and Sanger sequencing results confirm HPV18‐E7 oncogene disruption and a higher gene disruption rate compared with Lipofectamine 2000 indicates that our micellar carrier is more advantageous (Figure 3D; Figure S7B, Supporting Information). Interestingly, we found large insertions in both the groups treated with gRNAs E71 and E72, and the inserted sequences were from the pCas9 backbone itself (Figure 3E; Figure S6A,B, Supporting Information). Because there is no sequence homology between pCas9 backbone and the E7 oncogene region, and the insertion was not found on the group treated with Lipofectamine, our carrier might play a role in boosting gene insertion. The mechanism behind this is unclear, and a detailed study is in progress to gain a better understanding.

On the other hand, the transient Cas9 expression profile on the F127/PPO‐NMe_3_/pCas9 micelle leads to a reduced off‐target effect. Earlier works have studied the specificity of the gRNA design. Although Cong et al. reported highly specific gRNA targeting and abolished gRNA function, caused by mismatches at the last 11 bases on the 20‐mer‐targeting region,^2^ subsequent work indicated that any mismatches on the 20‐mer‐targeting region of gRNA might cause Cas9 off‐targeting.^29^ With the optimized micelle, no significant editing was observed in the off‐targeting sites identified by both Cas‐OFFinder and BLAST (off/on ratio ≈0; Figure 3F,G). This establishes that our micelle has lower off‐target effect because of its transient transfection kinetics and the high gene‐targeting specificity of the CRISPR/Cas9 system.

While the findings with pCas9 suggest the proposed micelle's potential for genome editing applications, the intrinsic sequence preference of Cas9 limits the fidelity of this therapeutic approach. *Streptococcus pyogenes* Cas9 prefers targets with a PAM motif of NGG, which is difficult for AT‐rich sequence targeting.^2^ Although several Cas9 variants have been developed to tackle this issue, for example, *Acidaminococcus sp*. BV3LC Cpf1 can target the AT‐rich region as its PAM motif preference of TTTV (V = A, C, or G),^4^ the field is still looking for a more universal solution. Prokaryotic Argonaute proteins are potential options because they have much lower sequence preference. *Thermus thermophilus* Argonaute is one example, but it only functions at a relatively high temperature (75 °C).^30^ NgAgo was the first reported Argonaute system that could digest dsDNA at 37 °C.^13^ Inspired by the report that NgAgo could perform gene editing functions guided by DNA, we therefore explored the possibility to deliver NgAgo, which may allow for higher flexibility on gene targeting, using our micelle. However, after detailed studies in our lab and reported by other groups,^14^ we could not reproduce the gene editing function of NgAgo. Instead, it may function as an RNA effector.^15^ The optimized micelle is able to deliver NgAgo and interferes with the HPV18‐E7 expression in HeLa cells. Compared with the typical siRNA action, NgAgo acts as a single component, multi‐turnover RNA effector; it is also not involved in any intrinsic RNAi pathway. Theoretically, this system may be more efficient on gene silencing. In this study, we observed an effective knockdown of 22.9% efficiency at a gDNA dosage as low as 10 ng (1.4 pmol) at 48 h post‐gDNA transfection (Figure 4A). This gene knockdown also consequently affects the downstream proteasome activity and cell proliferation rate (Figure 4B,C). However, because of the gene knockdown observed with gDNA alone at the high dosage of 500 ng, we cannot be certain that NgAgo possesses the RNA interference effect. It suggests that gDNA alone may affect the gene expression under the antisense oligonucleotide mechanism (Figure 4D).

In the in vivo setting, our micelle was able to transfect the HeLa xenografted tumor with E7‐targeting pCas9 and delayed the tumor growth without affecting the physiological conditions of the mice (Figure 5A–C; Figure S16, Supporting Information). The histological analysis confirms the observation on tumor inhibition. E7 oncoprotein expression was reduced, and the downstream tumor suppressor Rb was rescued by the micelle‐delivered, E7‐targeting pCas9 (Figure 5D–F). From the Sanger sequencing analysis, out of 36 randomly selected colonies obtained from the tumor tissue treated with micelle‐delivered Cas9 and gRNA E72, we detected four Cas9‐induced mutations (Figure 5G). Although the editing efficiency in vivo (4/36, 11.1%) is lower than the in vitro efficiency (8/23, 34.8%), our micelle still shows at least 3.7‐fold improvement on gene editing using nonvirally delivered Cas9, when compared with the recently reported Cas9 plasmid delivery system demonstrating 3% gene disruption rate.^31^

By contrast, micelle‐delivered NgAgo did not show significant effects on tumor inhibition (Figure 6A; Figure S18C, Supporting Information), but only slight reduction in E7 expression in the extracted tumor tissues (Figure 6B). Moreover, unlike Cas9 treatment, no obvious difference between groups was detected on the histological samples with E7‐staining, Rb‐staining, or even H&E‐staining (Figure 6C–E). The reason causing the discrepancy between the two systems could be their different gene manipulation mechanisms. Further studies would be needed to clarify the mechanism of NgAgo's RNA interference and the interaction between NgAgo–gDNA complex and the RNA target. In addition, other similar RNA‐guided riboendonucleases, such as Cas13a/C2c2^32^ and Csm,^33^ were recently reported for RNA silencing application. Those enzymes including NgAgo may serve as potential alternatives to RNAi, but more studies, especially more in vivo characterization, would be needed.

In summary, we designed and optimized a F127/PPO‐NMe_3_ micelle for plasmid‐based Cas9 and NgAgo delivery. This micelle enables a more transient protein expression, which reduces potential Cas9 off‐targeting. Compared with other gene carriers, the micelle optimized in this study is colloidally more stable, less toxic, and more potent in both Cas9 and NgAgo deliveries. For both in vitro and in vivo HPV models, this micelle efficiently delivered Cas9 plasmid and disrupted the HPV18‐E7 oncogene to suppress the cancer progression. In addition to Cas9 delivery, the micelle was also capable of delivering NgAgo for gene manipulation. However, no signification E7 inhibition was observed in vivo, although the micelle‐delivered NgAgo may interfere with E7 expression and regulated its downstream pathway in vitro. Nevertheless, these findings suggest the promise of this F127/PPO‐NMe_3_ micellar carrier for therapeutic gene manipulation.

## 4 Experimental Section

*Chemicals*: Poly(propylene oxide) bis(2‐aminopropyl ether) (PPO‐NH_2_, average *M*_n_ 4000), Pluronic copolymer F127, PEI, and methanol were purchased from Sigma‐Aldrich (St Louis, MO) and used without further purification. Dichloromethane, iodomethane, potassium carbonate, and 3‐(4,5‐dimethyl‐thiazol‐2‐yl)‐2,5‐diphenyl tetrazolium bromide (MTT) were obtained from Alfa Aesar (Tewksbury, MA).

*Synthesis of PPO‐NMe_3_*: PPO‐NMe_3_ was synthesized through the quaternization of PPO‐NH_2_ (Figure 1B). PPO‐NH_2_ (2.0 g, 0.5 mmol) was first dissolved in dichloromethane/methanol (3/1, v/v) and then potassium carbonate (1.378 g, 1.0 mmol) was gently added. The mixture was mixed at room temperature (RT) for 30 min, followed by an addition of iodomethane (9.35 mL, 150 mmol). The resulting mixture was further heated at reflux for 48 h under a dry nitrogen atmosphere. After cooling to RT, the mixture was filtered, and the solvents were removed by rotary evaporation. The given oily product was further purified by dialysis against 0.5% NaCl solution (48 h) and distilled water (72 h). The final product was obtained in the form of an oily liquid after lyophilization of the dialyzed solution. ^1^H NMR spectrum of the product was recorded on Bruker AV 400 NMR spectrometer (Billerica, MA) in DMSO‐*d*_6_, with tetramethylsilane as the internal standard.

*Preparation of F127/PPO‐NMe_3_ Micelle*: F127 and the nucleic acid (pCas9, pNgAgo–EGFP, or gDNAs) were mixed in a desired w/w ratio by gentle vortexing. After 5 min incubation at RT, PPO‐NMe_3_ was added, and the mixture was vortexed again, followed by a transfection process or other characterizations.

*Size and Zeta Potential Measurements*: Particle size and zeta potential were measured using ZetaSizer NanoZS‐90 (Malvern Instruments, Southborough, MA), with a fixed pCas9 (w/o gRNA) concentration at 1 µg mL^−1^. For size measurement, the scattering angle was fixed at 90°. To evaluate the stability of the complexes in Opti‐MEM (Thermo Fisher, Waltham, MA), size measurements were conducted at 30 min intervals over a 4 h period. Zeta‐potential measurements were performed using a capillary flow cells in water.

*All‐in‐One pCas9 Construct Preparation*: The all‐in‐one pCAS9 constructs were established by following the previously published protocol^21^ without a significant revision. The gRNA‐encoding dsDNAs for cloning were synthesized by IDT (Coralville, IA). The cloned plasmids were transformed into One Shot Stbl2 competent cells (Thermo Fisher) and then purified using NucleoBond Xtra Midi Plus EF kit (Clontech, Mountain View, CA). The gRNA sequences were verified by Eton Bioscience (Union, NJ) with the LKO‐1 5′ primer (listed in Table S1, Supporting Information).

*Cell Culture, Cytotoxicity, and Transfection*: HeLa cells were maintained in the complete medium, composed of DMEM (Thermo Fisher, Waltham, MA) with 10% FBS (Atlanta Biologicals, Flowery Branch, GA), 100 U mL^−1^ of penicillin–streptomycin (Thermo Fisher), and 1 × MEM Non‐Essential Amino Acids (Thermo Fisher), at 37 °C in a humidified atmosphere containing 5% CO_2_.

The cytotoxicity of F127/PPO‐NMe_3_/pCas9 complexes was evaluated by MTT assay against HeLa cells. Briefly, HeLa cells were first seeded in a 96‐well plate (2 × 10^4^ cells per well) for 24 h. The culture medium was replaced with Opti‐MEM containing F127/PPO‐NMe_3_/pCas9 complexes, followed by changing the medium back to complete medium after 4 h. After another 24 h incubation, the cells were subjected to the MTT assay. The absorbance of the solution was measured with BMG Lab‐tech FLUOStar Optima microplate reader (Germany) at 570 nm.

For transfection, the cells were seeded in a 24‐well plate (7.5 × 10^4^ cells per well) one day prior to the transfection. The transfection was carried out in Opti‐MEM. After 4 h incubation, the Opti‐MEM was replaced by the complete medium for further culture. The transfection efficiency was determined using BD LSR Fortessa flow cytometer (San Jose, CA). In general, the transfected cells were harvested in complete media, and Cas9‐GFP^+^ cells were gated based on the florescence intensity on the FITC channel of the flow cytometer. The results were analyzed by FlowJo 7.6.1 (FlowJo, LLC, Ashland, OR).

*Confocal Laser Scanning Microscopy (CLSM) Measurement*: HeLa cells were transfected in a 24‐well plate by following the transfection protocol as aforementioned. At 24 h post‐transfection, cells were washed three times with phosphate‐buffered saline (PBS) and fixed with 4% formaldehyde. Then, the cells were counterstained with 4′,6‐diamidino‐2‐phenylindole (DAPI) and Alexa Fluor 647‐labeled phalloidin (Thermo Fisher) for nucleus and actin staining, respectively, following the manufacturer's instructions, and then visualized under the laser scanning confocal microscope (ZEISS LSM 800, Germany).

*Downstream Proteasome Assay*: HeLa cells were seeded and transfected in a 96‐well plate first. After 72 h of incubation, the cells were subjected to proteasome and MTT assays. For the proteasome assay, the cells were washed once with PBS and then incubated with 1 × Proteasome assay buffer containing the proteasome substrates (Sigma‐Aldrich) at 37 °C for 2 h. After incubation, the supernatant was transferred to a 96‐well opaque plate, and the fluorescence was detected using BMG Biotech FLUOstar OPTIMA microplate reader with a 490 nm band pass excitation and 520 nm band pass emission filters. Data were normalized to the fluorescence intensity of the group treated only with Cas9 or NgAgo plasmid.

*Gene Disruption Validation*: To validate the gene disruption, the transfected HeLa cells were sorted with the GFP marker. At 24 h post‐transfection, the cells were harvested in Opti‐MEM with a concentration of ≈2 × 10^7^ cells mL^−1^. In addition, DNase I (10 U per sample; Thermo Fisher) was added to prevent cell aggregation. Samples were then filtered and sorted using BD Influx Cell Sorter under the assistance of the staff in the Flow Cytometry Core Facility, Columbia Center for Translational Immunology (CCTI). GFP^+^ cells were collected in a 96‐well plate (1.5 × 10^4^ cells per well) in complete media for further culture and validation.

The sorted cells were harvested and lysed using Clontech Guide‐it Mutation Detection kit. The Cas9‐targeting locus was amplified using 2‐step PCR (98 °C for 10 s and 68 °C for 1 min) with Clontech Terra Polymerase (1.25 U per sample) and 0.3 × 10^−6^ m of the primers (all the primers listed in Table S1, Supporting Information) for 35 cycles. After amplification, PCR amplicon was purified with NucleoSpin Gel and PCR Clean‐up column (Clontech). DNA concentration was determined by UV–VIS.

For the T7 endonuclease I assay, 200 ng of the PCR product was reannealed in 1 × NEB buffer 2 (New England Biolabs, Ipswich, MA) to form mismatched heteroduplexes via a temperature gradient process. The reannealed sample was subsequently incubated with 10 U of T7 endonuclease I (New England Biolabs) for 15 min at 37 °C. Then, HPV oncogene disruption was verified on an ethidium bromide‐prestained 2% TAE‐agarose gel.

For mutation identification, the PCR product was cloned into a pUC19 vector (Clontech) and transformed to the Clontech Stellar competent cells by following manufacturer's protocol. After overnight culture of the transformed bacteria, the clone was picked up and directly amplified using the same 2‐step PCR for 30 cycles. The PCR product was verified using gel electrophoresis and sequenced by Eton Bioscience with the primer pUC19_SEQ_F (listed in Table S1, Supporting Information).

*HPV E7 Gene Knockdown with Micelle‐Delivered NgAgo*: NgAgo was first delivered to HeLa cell with the optimized micelle or Lipofectamine. At 24 h post‐pNgAgo transfection, the cells were transfected with the corresponding gDNAs using the same transfection protocol. After another 48 h incubation, the cells were lysed with Trizol reagent (Thermo Fisher), and the total mRNAs were extracted using Zymo Research Direct‐zol MiniPrep Plus kit (Irvine, CA) with DNase I treatment. The total mRNAs were quantitated by UV–VIS and converted to cDNA using Bio‐Rad iScript cDNA synthesis kit (Hercules, CA).

To quantify the HPV18‐E7 gene expression level, the cDNA product was amplified and monitored real‐time on a Thermo Fisher StepOne Plus PCR machine. Each reaction mix contained 20 ng of the cDNA products, 0.25 × 10^−6^ m of primers (all the primers listed in Table S1, Supporting Information), 1 × Bio‐Rad SsoAdvanced Universal SYBR Green reagent. The reaction was carried out under a thermal cycling process (98 °C for 10 s, 60 °C for 60 s) with an initial heat‐activation step (98 °C for 30 s). To calculate the knockdown efficiency, the HPV18‐E7 gene expression level was obtained by normalization to the internal glyceraldehyde 3‐phosphate dehydrogenase (GADPH) control, and the result was then normalized to that of the group treated with only NgAgo without gDNA.

*In Vivo Transfection and Antitumor Effect Validation*: All the animal studies were performed following the protocol approved by the Institutional Animal Care and Use Committee of Columbia University. Female nude (nu/nu) mice in the 4–6 week age range were purchased from Charles River (Wilmington, MA). To establish the xenograft model, each mouse was subcutaneously injected with HeLa cells suspended in PBS. For Cas9 validation, when the tumor size reached ≈100 mm^3^, PBS (*n* = 5), carrier (F127/PPO‐NMe_3_ micelle w/o plasmid; *n* = 5), or pCas9‐loaded micelle (w/o gRNA, with gRNA E71 or E72; *n* = 6 for each group) was administered intratumorally with a dosage equivalent to 5 µg of the plasmid every four days for a month. Tumor volume and body weight of each mouse were recorded every two days. The tumor volume was calculated based on the length of its long and short axes: volume = 1/2 × long axis length × (short axis length)^2^.

After the treatment, the mice were sacrificed, and tumors were extracted. A portion of the tumor tissue (≈25 mg) was directly digested in Zymo Research Solid Tissue Buffer supplemented with protease K (1 mg mL^−1^) at 55 °C for overnight, and the genomic DNAs were then extracted using Zymo Research Quick‐DNA Miniprep Plus kit. Using the same protocol for in vitro mutation identification, the target HPV18‐E7 locus was subsequently amplified, and the Cas9‐induced mutations were detected by Sanger sequencing. On the other hand, the rest of tumor tissue and the major organs (lung, heart, liver, spleen, kidney) were fixed in 4% paraformaldehyde for 24 h and dehydrated in 70% ethanol. The tissues were processed by the Molecular Pathology Core of Herbert Irving Comprehensive Cancer Center at Columbia Medical Center for H&E, HPV18 E7, and Rb staining.

The NgAgo validation was carried out with the same subcutaneous xenograft setting. When the tumor reached ≈100 mm^3^, the treatment course was given every 4 d for a month. For each course, all the mice were administered intratumorally with pNgAgo–EGFP‐loaded micelle first, and two days later, PBS, gDNA E71, or E72 was given using the optimized micelle through the same local route (*n* = 5 for each group). Each injection contains either pNgAgo–EGFP or gDNA with an equivalent amount of 5 µg. After the treatment, part of the tumor tissue was extracted, and the total RNAs were obtained via direct tissue homogenization in TRIzol reagent. The RNAs were further purified and quantitated with the aforementioned protocol. Similar to Cas9 validation, the rest of the tumor tissues and major organs were also fixed and processed for histochemical and immunohistochemical staining.

## Conflict of Interest

The authors declare no conflict of interest.

## Supporting information

Supplementary
