# Supplementary material for: HPV Oncogene Manipulation Using Nonvirally Delivered CRISPR/Cas9 or Natronobacterium gregoryi Argonaute
Source: Adv Sci (Weinh). 2018 May 18;5(7):1700540. doi: 10.1002/advs.201700540 (PMC6051382; doi:10.1002/advs.201700540)
Supplement: Supplementary file 1 — Supplementary [file ADVS-5-1700540-s001.pdf]

Copyright WILEY-VCH Verlag GmbH & Co. KGaA, 69469 Weinheim, Germany, 2018.

ADVANCED SCIENCE
Open Access

Supporting Information

for *Adv. Sci.,* DOI: 10.1002/advs.201700540

HPV Oncogene Manipulation Using Nonvirally Delivered CRISPR/Cas9 or *Natronobacterium gregoryi* Argonaute

*Yeh-Hsing Lao, Mingqiang Li, Madeleine A. Gao, Dan Shao, Chun-Wei Chi, Dantong Huang, Syandan Chakraborty, Tzu-Chieh Ho, Weiqian Jiang, Hong-Xia Wang, Sihong Wang, and Kam W. Leong**

WILEY-VCH

ADVANCED SCIENCE Open Access

Copyright WILEY-VCH Verlag GmbH & Co. KGaA, 69469 Weinheim, Germany, 2016.

Supporting Information

HPV oncogene manipulation using non-virally delivered CRISPR/Cas9 or *Natronobacterium gregoryi* Argonaute

Yeh-Hsing Lao,[1†] Mingqiang Li,[1†] Madeleine A. Gao,[1] Dan Shao,[1] Chun-Wei Chi,[2] Dantong Huang,[1] Syandan Chakraborty,[1] Tzu-Chieh Ho,[1] Weiqian Jiang,[1] Hong-Xia Wang,[1] Sihong Wang,[2] and Kam W. Leong[1,3]*

[1] Department of Biomedical Engineering, Columbia University, New York NY 10027
[2] Department of Biomedical Engineering, CUNY- City College of New York, New York, NY 10031
[3] Department of Systems Biology, Columbia University Medical Center, New York NY 10032
* corresponding author: Prof. Kam W. Leong (kam.leong@columbia.edu)
† these authors contributed equally to this work.

This file includes:

Supplementary Materials and Methods
Figure S1. Optimization of micelle formulation.
Figure S2. Characterization of the optimized micelle.
Figure S3. Enzymatic degradation evaluation for the pCas9-encapsulated micelle.
Figure S4. Cellular uptake of pCas9-encapsulated micelle.
Figure S5. Sequence verification of the pCas9 constructs used in this study.
Figure S6. Sequencing validation of the micelle-delivered CRISPR/Cas9.
Figure S7. HPV18-E7 knockout using Lipofectamine 2000.
Figure S8. Potential off-target sites of the gRNAs used in this study.
Figure S9. FLAG-NgAgo-NLS expressing HeLa cell line generation and HPV18-E7 oncogene knockout validation.
Figure S10. HPV18-E7 knockdown in the FLAG-NgAgo-NLS expressing HeLa cell.
Figure S11. Plasmid map and the NgAgo CDS of the pNgAgo-EGFP construct established in study.
Figure S12. Physicochemical characterization of pNgAgo-EGFP-encapsulated micelle.
Figure S13. Cellular uptake of pNgAgo-encapsulated micelle.
Figure S14. Validation on real-time PCR result variation.
Figure S15. HPV18-E7 gene knockdown with Lipofectamine-delivered NgAgo.
Figure S16. H&E staining for the major organs extracted from the pCas9-micelle-treated mice.
Figure S17. Sequencing validation of the tumor extracted from the Cas9 control group.
Figure S18. *In vivo* evaluation of micelle-delivered NgAgo.
Table S1. Sequences of the primers and gDNAs used in this study.

Supplementary Materials and Methods

DNase I digestion assay

The naked pCas9 plasmid or pCas9-encapsulated micelle (equivalent plasmid concentration = 10 ng/μL) was incubated with DNase I (Thermo Fisher) under physiological DNase I condition (DNase I activity = 1.1×10^{-3} mg min^{-1} mL^{-1})[1] at 37°C for 0, 2, 4, 8 or 24 h. After the incubation, DNase I was inactivated by heating the samples at 95°C for 10 min. All the samples were subsequently incubated with heparin (20 mg/mL) at 37°C for 30 min and run on a 1.5% ethidium bromide-prestained TAE-agarose gel directly.

Lentiviral FLAG-NgAgo-NLS plasmid construction

NgAgo expression in the lentiviral plasmid is controlled by a tetracycline-inducible promoter. To construct the FLAG-NgAgo-NLS lentiviral plasmid, both NLS-NgAgo-pCDNA3.1 (Miaoling Bio, China) and Tet-O-FUW-Myt1l (Addgene #27152) were first digested with NheI-HF and EcoRI-HF (NEB) restriction enzymes to produce compatible sticky ends between the NLS-NgAgo fragment and the Tet-O-FUW backbone, both of which were extracted from an 1% agarose gel using QIAquick gel extraction kit. The Tet-O-FUW backbone was further dephosphorylated with Antarctic phosphatase (NEB). The two fragments were subsequently ligated using T4 ligase (NEB). The ligated product was transformed to the Stbl3 competent cells (Thermo Fisher), and the plasmid was extracted using Macherey-Nagel NucleoSpin® plasmid purification kit (Germany).

Lentivirus production was performed in HEK 293T cells cultured in a T75 flask by introducing pFLAG-NgAgo-NLS, psPAX2 (Addgene #12260) and pMD2.G (Addgene #12259) at the 3:2:1 mass ratio, to the cells in the presence of CalFectin (SignaGen Laboratories, Rockville, MD), based on the manufacturer's recommendation. Cells were replenished with fresh medium 16 hours post-transfection. Media containing viruses were collected at 40 h and 64 h post-transfection and concentrated using centrifugal filter units with 100 kD membrane (EMD Millipore).

FLAG-NgAgo-NLS-expressing HeLa stable cell line generation

HeLa cells were first seeded in 6-well plate with a density of 2×10^5 cells per well in 2.5 mL of DMEM and incubated for 24 h. For transduction, the cells were incubated with the reverse tetracycline-controlled transactivator-carrying and FLAG-NgAgo-NLS-carrying lentiviruses with a total MOI of 50, and polybrene (8 µg/mL) was used to enhance the efficiency. To further improve the efficiency, the transduction was repeated 3 times in a 3-day schedule. The transduction efficiency was evaluated by immunostaining. Briefly, the transduced HeLa cells were first seeded in a 24-well plate, and FLAG-NgAgo-NLS expression was induced using doxycycline (3 µg/mL). After 2-day doxycycline induction, the transduced cells were fixed and permeabilized. The cells were subsequently stained with anti-FLAG tag antibody (Thermo Fisher) and the secondary antibody, Alexa Fluor® 488 donkey anti-mouse IgG (Thermo Fisher), for immunofluorescent visualization. The cells were also stained with DAPI to visualize the nucleus. Fluorescent images were taken using Nikon Eclipse TE2000-U inverted microscope.

Non-viral NgAgo-EGFP plasmid construction

The mammalian codon-optimized NgAgo coding sequence (CDS) was obtained from the NLS-NgAgo-pCDNA3.1 construct by PCR using NEB heat-activated Q5 DNA polymerase, and the EGFP-FLAG vector was purchased from Addgene (#46956). For pNgAgo-EGFP construction, the vector was first digested with the NEB EcoRI-HF restriction enzyme at 37°C for 1h and then purified by Thermo Fisher PureLink DNA purification kit. Subsequently, the linear vector was blunted and de-phosphorylated using NEB's recommended protocols with Klenow polymerase and Antarctic phosphatase, respectively. The blunted, de-phosphorylated vector was ligated with the purified NgAgo CDS PCR product (insert/vector molar ratio = 3) using NEB T4 ligase at 16°C for overnight. Followed by the ligation, the plasmid was transformed to the Clontech Stellar competent cells by following manufacturer's protocol. Singe clone was picked and further amplified in a large-scale culture for overnight at 37°C. The plasmid was purified using

Macherey-Nagel NucleoBond® Xtra Midi Plus EF plasmid purification kit, and the sequences were verified by both PCR and Eton Bioscience (Union, NJ).

Transmission electron microscopy (TEM) measurement

The pCas9- and pNgAgo-EGFP- encapsulated micelles were negatively stained with 5% phosphotungstic acid (Electron Microscopy Sciences, Hatfield, PA). TEM measurement to visualize the micelles was then performed using FEI Talos F200X transmission electron microscope under an accelerating voltage of 200 KV.

Reference

[1] W. S. Prince, D. L. Baker, A. H. Dodge, A. E. Ahmed, R. W. Chestnut, D. V. Sinicropi, *Clin Exp Immunol* **1998**, 113, 289.

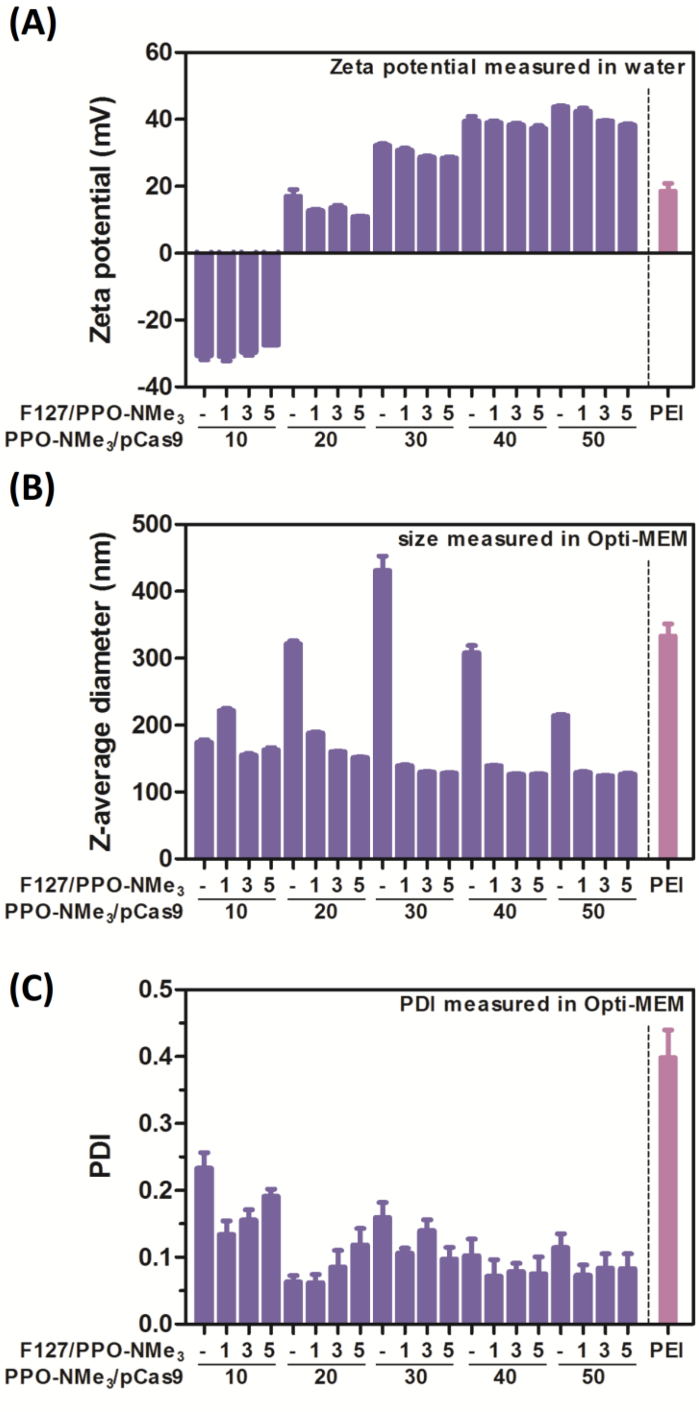

Figure S1. Optimization of micelle formulation. (A) Zeta potential, (B) size and (C) PDIs of the F127/PPO-NMe_3/pCas9 micelle in different compositions. Data are presented as average ± SEM (n = 4).

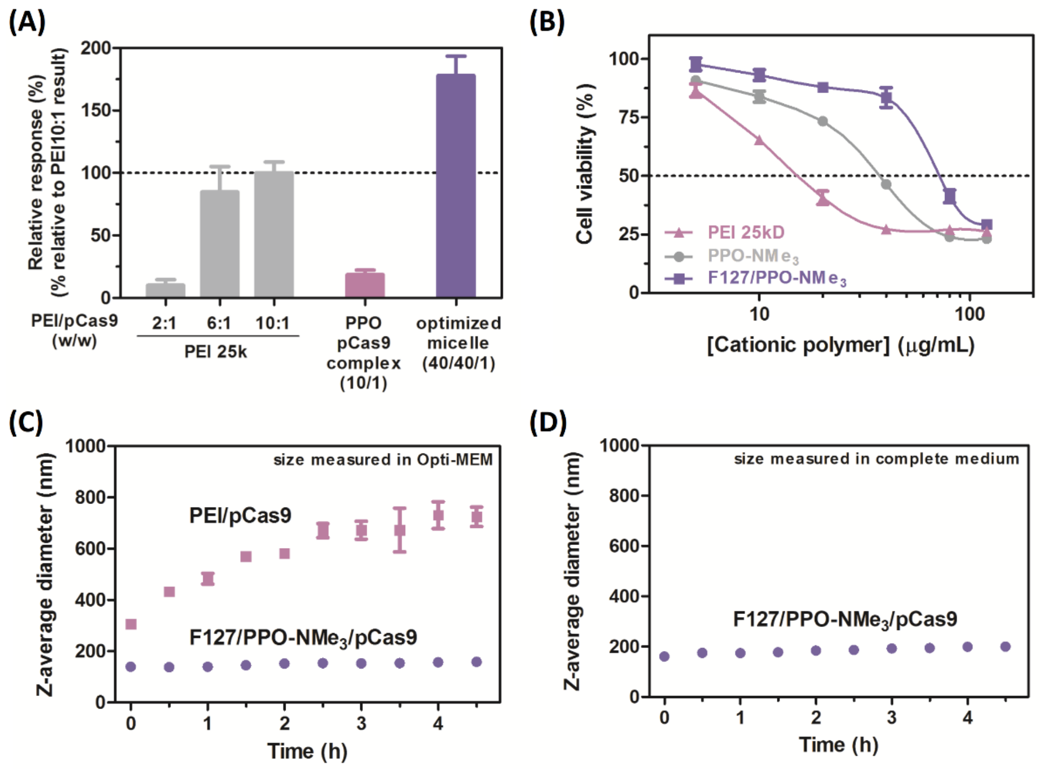

Figure S2. Characterization of the optimized micelle. (A) Relative Cas9 transfection efficiencies of PEI in different polymer/pCas9 ratios. The transfection efficiency was determined using FACS based on the GFP signal. Relative efficiency was calculated *via* normalization to the efficiency of PEI with the polymer/pCas9 ratio of 10/1. Data are presented as average ± SEM (n = 5). (B) *In vitro* cytotoxicity of PEI/pCas9, PPO-NMe_3/pCas9 and F127/PPO-NMe_3/pCas9 in HeLa cells. Cytotoxicity was determined by MTT assay. Data are presented as average ± SEM (n = 3). (C) Stability of PEI/pCas9 and F127/PPO-NMe_3/pCas9 micelle in Opti-MEM. (D) Stability of F127/PPO-NMe_3/pCas9 micelle in complete medium (with 10% FBS). Data are presented as average ± SEM (n = 4).

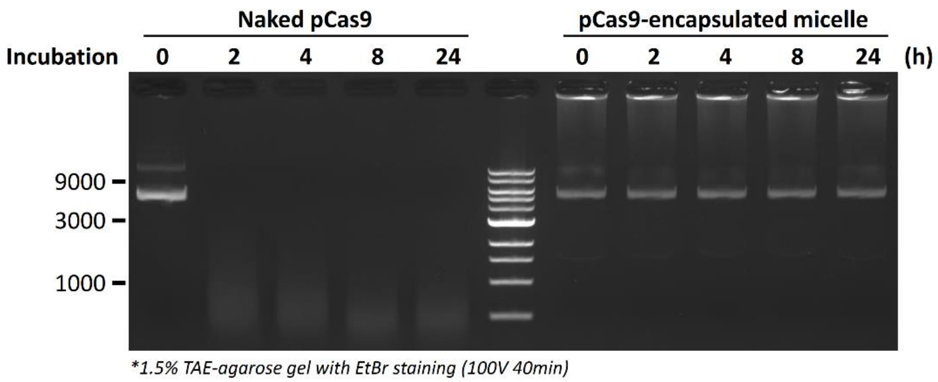

Figure S3. Enzymatic degradation evaluation for the pCas9-encapsulated micelle. The naked pCas9 plasmid or pCas9-encapsulated micelle was incubated with DNase I under the physiological condition for 0, 2, 4, 8 or 24 h. Afterwards, DNase I was inactivated, and the samples were further incubated with heparin at 37°C. The plasmid and its degraded products were visualized on a 1.5% TAE-agarose gel stained with ethidium bromide.

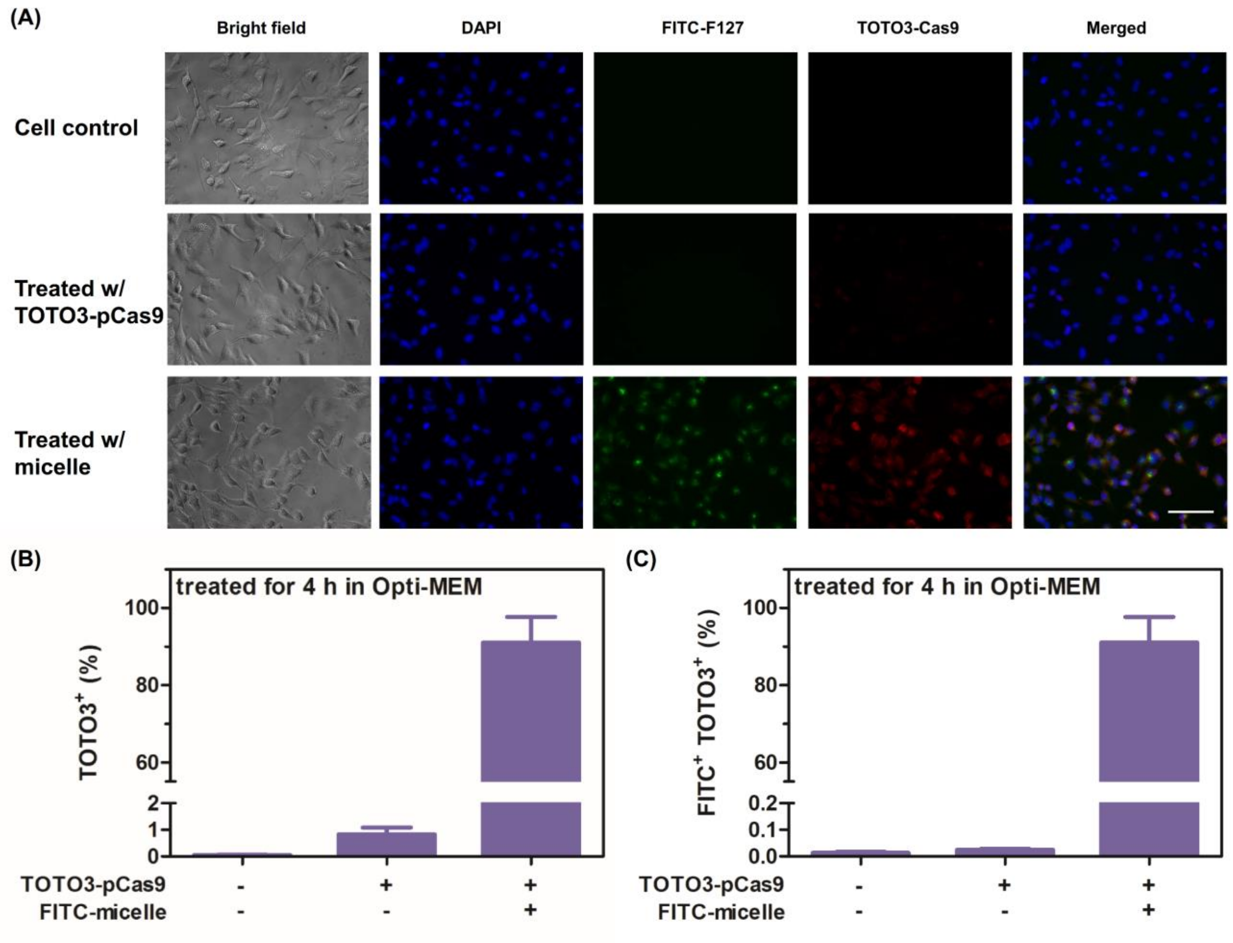

Figure S4. Cellular uptake of pCas9-encapsulated micelle. (A) Fluorescent images of the HeLa cells treated with FITC-F127/PPO-NMe_3/TOTO3-pCas9 micelle for 4 h. Scale bar represents 100 μm. (B) Total plasmid-accessible cell % and (C) micelle-mediated plasmid-accessible cell %, quantified using FACS. For the cellular uptake study, cells were seeded in a 24-well plate one day prior to transfection. The Cas9 plasmid (Addgene #62934) was first stained with TOTO3 under a base-pair/dye molar ratio of 10/1 and complexed with PPO-NMe_3 and FITC-F127. Cells were treated with the micelle for 4 h in Opti-MEM and then either fixed for microscope measurement or harvested for FACS analysis. Cellular uptake rate ($TOTO3^{+}$% and $FITC^{+}$ $TOTO3^{+}$ %) are presented as average ± SEM (*n=3*).

gRNA E71

NNGGAATGCGATTTCTTGGCTTTATATATCTTGTGGAAAGGACGAAACACC**GGAGCAATTAAGCGACTCAG**GTTTT
AGAGCTAGAAATAGCAAGTTAAAATAAGGCTAGTCCGTTATCAACTTGAAAAAGTGGCACCGAGTCGGTGCTTTTTT
GTTTTAGAGCTAGAAATAGCAAGTTAAAATAAGGCTAGTCCGTTTTTAGCGCGTGCGCCAATTCTGCAGACAAATGG
CTCTAGAGGTACCCGTTACATAACTTACGGTAAATGGCCCGCCTGGCTGACCGCCCAACGACCCCCGCCCATTGACG
TCAATAGTAACGCCAATAGGGACTTTCCATTGACGTCAATGGGTGGAGTATTTACGGTAAACTGCCCACTTGGCAGT
ACATCAAGTGTATCATATGCCAAGTACGCCCCCTATTGACGTCAATGACGGTAAATGGCCCGCCTGGCATTGTGCCCA
GTACATGACCTTATGGGACTTTCCTACTTGGCAGTACATCTACGTATTAGTCATCGCTATTACCATGGTCGAGGTGAGC
CCCACGTTCTGCTTCACTCTCCCCATCTCCCCCCCCTCCCCACCCCCAATTTTGTATTTATTTATTTTTTAATTATTTTGTG
CAGCGATGGGGGCGGGGGGGGGGGGGGGGAGGGGGGAGGGAAGGAAAAAGGGGGGCTAAGCCCGGAATG
GGGAAAGGCGGAGGAGGTGCGGGGGGGGCCAATCGCAAGCGGCCCACTCCCAAAGTTTCCTTTTATGGCGAA

gRNA E72

NNAATTCGATTTCTTGGCTTTATATATCTTGTGGAAGGACGAAACACC**GAAGAAAACGATGAAATAGA**GTTTTAGA
GCTAGAAATAGCAAGTTAAAATAAGGCTAGTCCGTTATCAACTTGAAAAAGTGGCACCGAGTCGGTGCTTTTTTGTT
TTAGAGCTAGAAATAGCAAGTTAAAATAAGGCTAGTCCGTTTTTAGCGCGTGCGCCAATTCTGCAGACAAATGGCTC
TAGAGGTACCCGTTACATAACTTACGGTAAATGGCCCGCCTGGCTGACCGCCCAACGACCCCCGCCCATTGACGTCA
ATAGTAACGCCAATAGGGACTTTCCATTGACGTCAATGGGTGGAGTATTTACGGTAAACTGCCCACTTGGCAGTACA
TCAAGTGTATCATATGCCAAGTACGCCCCCTATTGACGTCAATGACGGTAAATGGCCCGCCTGGCATTGTGCCCAGTA
CATGACCTTATGGGACTTTCCTACTTGGCAGTACATCTACGTATTAGTCATCGCTATTACCATGGTCGAGGTGAGCCCC
ACGTTCTGCTTCACTCTCCCCATCTCCCCCCCCTCCCCACCCCCAATTTTGTATTTATTTATTTTTTAATTATTTTGTGCA
GCGATGGGGGCGGGGGGGGGGGGGGGGCGGGGGAAAGGGAAGAAAAAAGGGGGGCCCCCCGGGCCGGG
GGAAAAGGGGGGTAAAACGGGGGGGGTCCGGCGGGGGGGGGAGGCCCATTGGGGTTTGGGGGGGGGCATG
GGGGGGGGGGGCCATTTCCGGGTTTGGGGGGAAAGAGGGGGGGGTTTTTGCCTGGCCGCCTTTTTACGTGACC
TCCTGACTATTGGAGCCTCCCGCAGAAAGGGTCCC

gRNA CTRL

NNNAATTTCGATTTCTTGGCTTTATATATCTTGTGGAAGGACGAAACACC**GGCGCTTTGAGGATCCAACA**GTTTTAG
AGCTAGAAATAGCAAGTTAAAATAAGGCTAGTCCGTTATCAACTTGAAAAAGTGGCACCGAGTCGGTGCTTTTTTGT
TTTAGAGCTAGAAATAGCAAGTTAAAATAAGGCTAGTCCGTTTTTAGCGCGTGCGCCAATTCTGCAGACAAATGGCT
CTAGAGGTACCCGTTACATAACTTACGGTAAATGGCCCGCCTGGCTGACCGCCCAACGACCCCCGCCCATTGACGTC
AATAGTAACGCCAATAGGGACTTTCCATTGACGTCAATGGGTGGAGTATTTACGGTAAACTGCCCACTTGGCAGTAC
ATCAAGTGTATCATATGCCAAGTACGCCCCCTATTGACGTCAATGACGGTAAATGGCCCGCCTGGCATTGTGCCCAGT
ACATGACCTTATGGGACTTTCCTACTTGGCAGTACATCTACGTATTAGTCATCGCTATTACCATGGTCGAGGTGAGCCC
CACGTTCTGCTTCACTCTCCCCATCTCCCCCCCCTCCCCACCCCCAATTTTGTATTTATTTATTTTTTAATTATTTTGTGC
AGCGATGGGGGCGGGGGGGGGGGGGGGGGGCGGGCAGAGAGAAGCAGAACGGGGGGCCCCCCGGGGCCAT
GGGGATAGGGAGAGGTAAAACGGCGGTGTTCGGCGAGGGGGGAAGGAAAATTAGGGTTTTTGGGGGAGGCAA
TGGGGGGGGGGCTTTTTTCCGGATTTGGGGGTATAGGGGGGGGGTTCTGGTATTATTAACCTTTTTTTTT

Figure S5. Sequence verification of the pCas9 constructs used in this study. The gRNA sequences are colored red, and the 20mer targeting region of each gRNA is underlined. Three plasmids were purified using Macherey-Nagel NucleoBond® Xtra Midi Plus EF plasmid purification kit (Germany), and the sequences were identified by Eton Bioscience (Union, NJ).

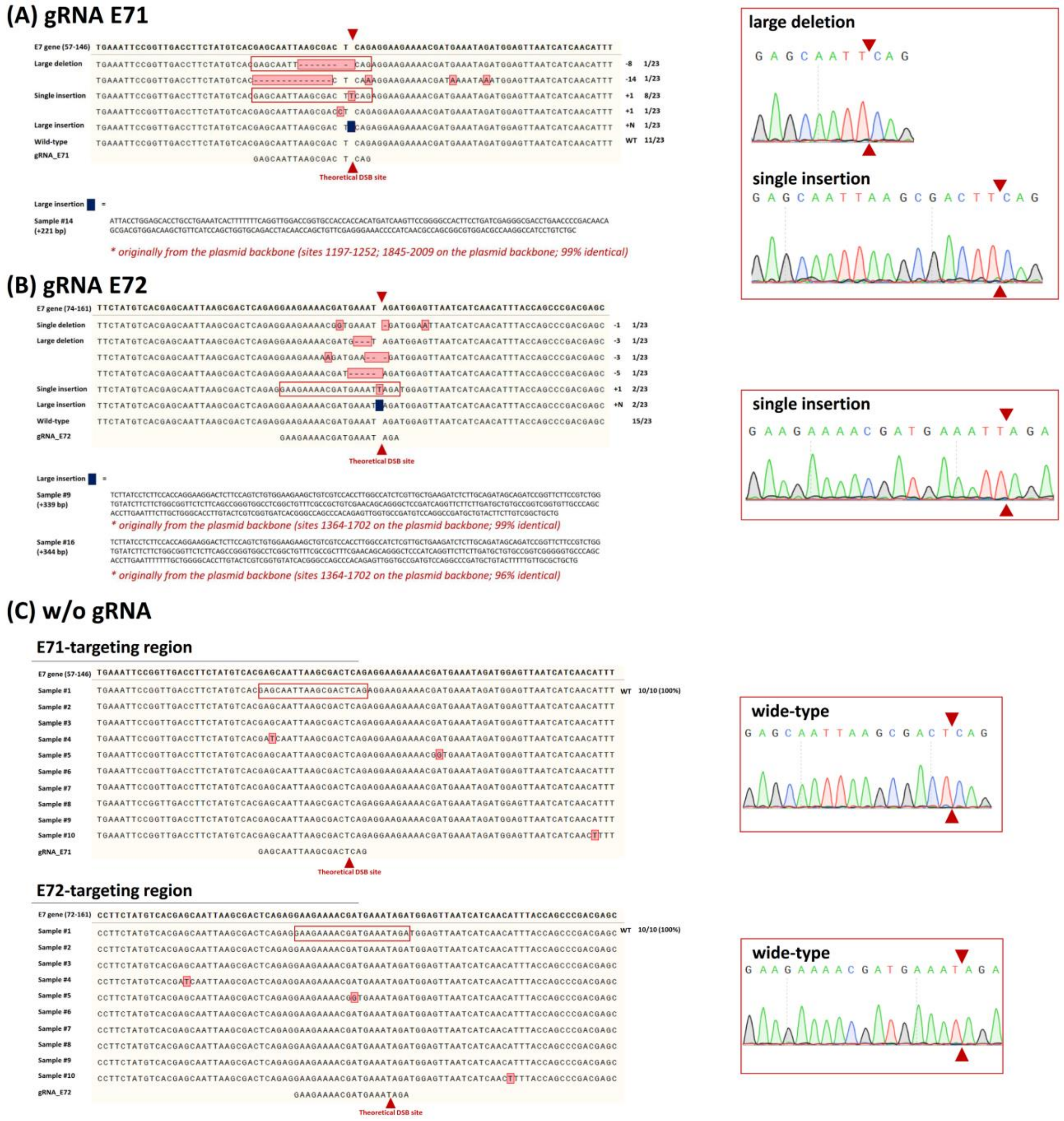

Figure S6. Sequencing validation of the micelle-delivered CRISPR/Cas9. Sequence alignments of the verified sequences from the HeLa cells transfected with (A) gRNA E71-, (B) gRNA E72-encoded pCas9 and (C) pCas9 w/o gRNA. Genomic DNA extraction, amplification and sequencing sample preparation were carried out with the protocol stated in the Experimental Section. Sequencing was done by Eton Bioscience with the primer pUC19_SEQ_F. (insets: representative sequencing results)

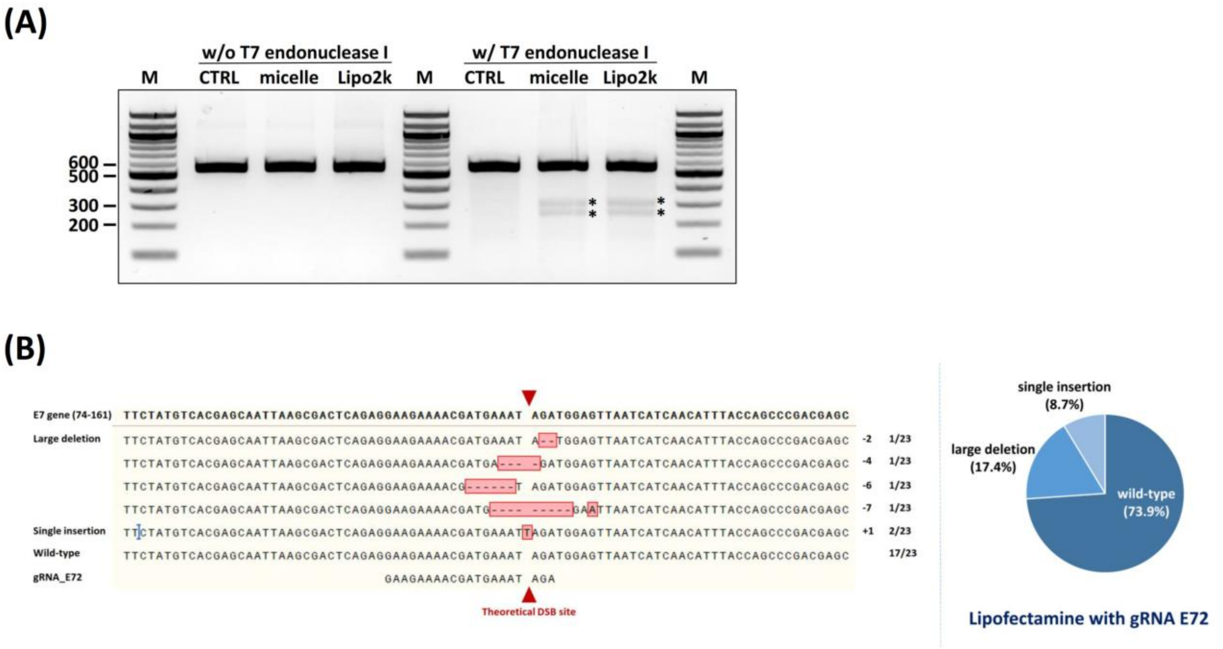

Figure S7. HPV18-E7 knockout using Lipofectamine 2000. (A) Gene editing efficiency comparison using T7EI assay (CTRL: w/o any transfection; micelle: micelle-transfected; Lipo2k: Lipofectamine 2000-transfected). (B) Sequencing validation of Lipofectamine-delivered CRISPR/Cas9. (Sequencing was done by Eton Bioscience with the primer pUC19_SEQ_F).

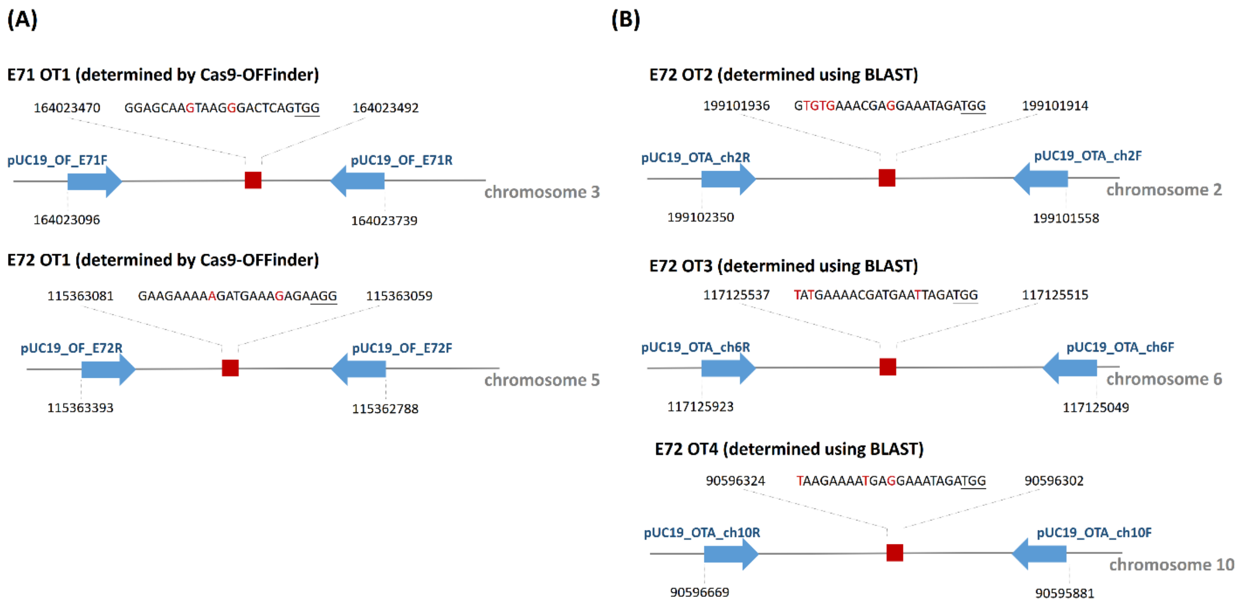

Figure S8. Potential off-target sites of the gRNAs used in this study. The off-target sites predicted using (A) Cas9-OFFinder and (B) BLAST. Mismatched bases and the PAM motif were colored red and underlined, respectively. The numbers represent the locations of each locus and its corresponding primer pair; these correspond to the locations in the chromosomes of GRCh38.p7 Primary Assembly. Primers were designed using NCBI Primer BLAST and listed in Table S1.

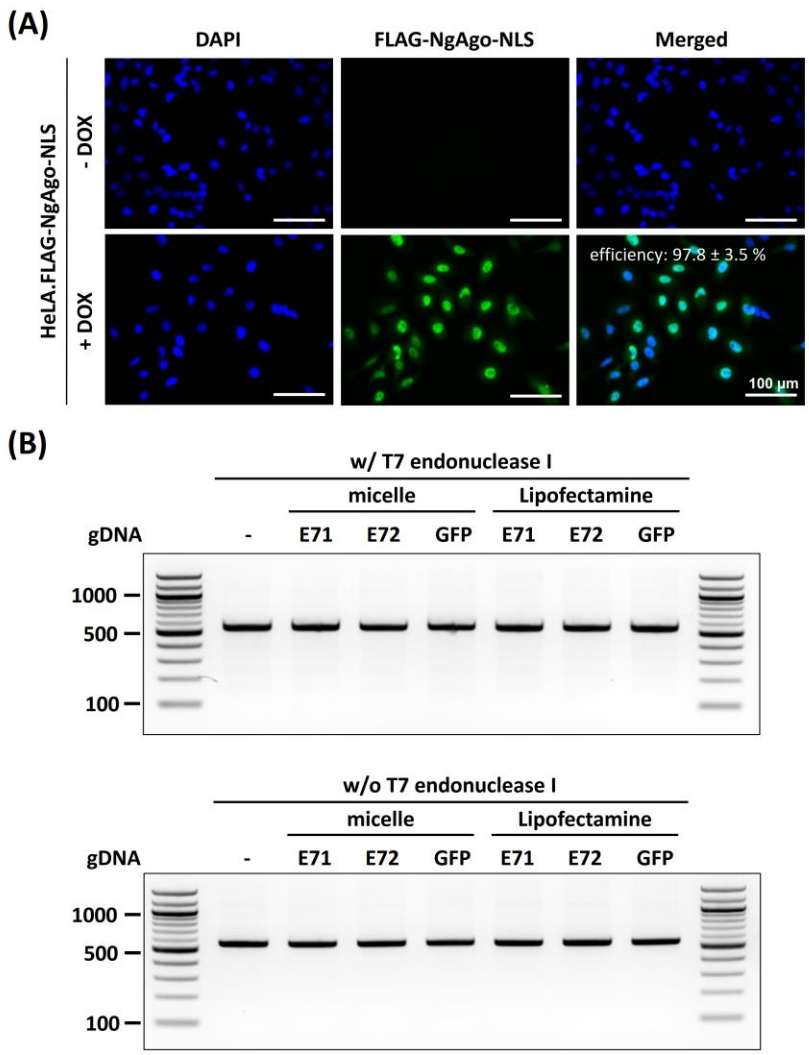

Figure S9. Validation on FLAG-NgAgo-NLS expressing HeLa cell line generation and HPV18-E7 oncogene knockout. (A) Immunofluorescent staining of FLAG-NgAgo-NLS stable line. The transduction efficiency was calculated from 20 individual images. (B) T7EI validation on the HPV18 E7 knockout. Virally-transduced HeLa cells were transfected with the gDNAs (E71, E72 or GFP-targeting) using either the optimized micelle or Lipofectamine 2000. At 48 h post-gDNA transfection, genomic DNAs were extracted, and the T7EI enzyme was used to detect NgAgo-induced mutations.

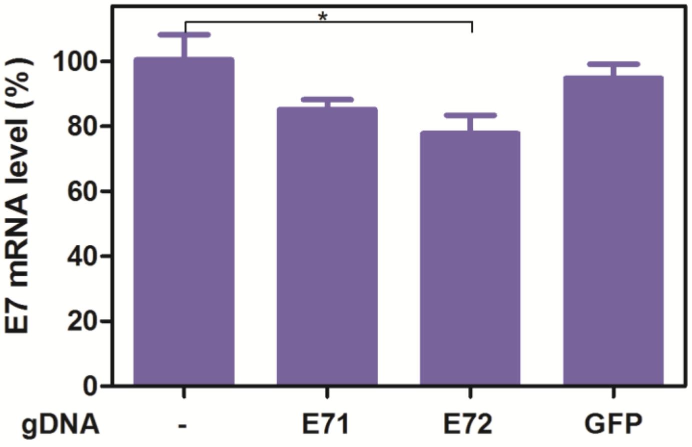

Figure S10. HPV18-E7 knockdown in the FLAG-NgAgo-NLS expressing HeLa cell. Virally-transduced HeLa cells were transfected with the gDNAs (E71, E72 or GFP-targeting) using Lipofectamine RNAiMax by following manufacturer's protocol. At 68 h post-gDNA transfection, total mRNAs were extracted, and RT-qPCR was carried out with the protocol mentioned in the Experimental Section. Data are presented as average ± SEM (n = 3). One-way ANOVA with Dunnett's multiple comparison test was used for p-value calculation. The significant level is represented as ∗ ($p < 0.05$).

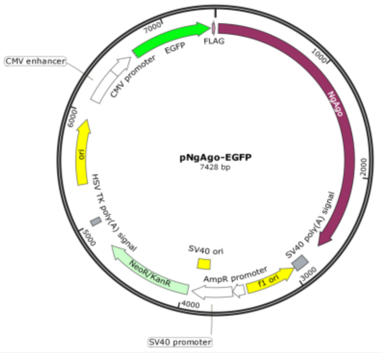

Codon-optimized NgAgo CDS

```
ACCGTGATCGACCTGGACAGCACCACCACCGCCGACGAGCTGACCAGCGGCCACACCTACGACATCAGCGTGACCCTGACCGGCGTGTACGACAACACCGACGAGCAGCA
CCCCCGCATGAGCCTGGCCTTCGAGCAGGACAACGGCGAGCGCCGCTACATCACCCTGTGGAAGAACACCACCCCCAAGGACGTGTTCACCTACGACTACGCCACCGGCAG
CACCTACATCTTCACCAACATCGACTACGAGGTGAAGGACGGCTACGAGAACCTGACCGCCACCTACCAGACCACCGTGGAGAACGCCACCGCCCAGGAGGTGGGCACCA
CCGACGAGGACGAGACCTTCGCCGGCGGCGAGCCCCTGGACCACCACCTGGACGACGCCCTGAACGAGACCCCCGACGACGCCGAGACCGAGAGCGACAGCGGCCACG
TGATGACCAGCTTCGCCAGCCGCGACCAGCTGCCCGAGTGGACCCTGCACACCTACACCCTGACCGCCACCGACGGCGCCAAGACCGACACCGAGTACGCCCGCCGCACCC
TGGCCTACACCGTGCGCCAGGAGCTGTACACCGACCACGACGCCGCCCCCGTGGCCACCGACGGCCTGATGCTGCTGACCCCCGAGCCCCTGGGCGAGACCCCCCTGGAC
CTGGACTGCGGCGTGCGCGTGGAGGCCGACGAGACCCGCACCCTGGACTACACCACCGCCAAGGACCGCCTGCTGGCCCGCGAGCTGGTGGAGGAGGGCCTGAAGCGC
AGCCTGTGGGACGACTACCTGGTGCGCGGCATCGACGAGGTGCTGAGCAAGGAGCCCGTGCTGACCTGCGACGAGTTCGACCTGCACGAGCGCTACGACCTGAGCGTGG
AGGTGGGCCACAGCGGCCGCGCCTACCTGCACATCAACTTCCGCCACCGCTTCGTGCCCAAGCTGACCCTGGCCGACATCGACGACGACAACATCTACCCCGGCCTGCGCG
TGAAGACCACCTACCGCCCCCGCCGCGGCCACATCGTGTGGGGCCTGCGCGACGAGTGCGCCACCGACAGCCTGAACACCCTGGGCAACCAGAGCGTGGTGGCCTACCAC
CGCAACAACCAGACCCCCATCAACACCGACCTGCTGGACGCCATCGAGGCCGCCGACCGCCGCGTGGTGGAGACCCGCCGCCAGGGCCACGGCGACGACGCCGTGAGCT
TCCCCCAGGAGCTGCTGGCCGTGGAGCCCAACACCCACCAGATCAAGCAGTTCGCCAGCGACGGCTTCCACCAGCAGGCCCGCAGCAAGACCCGCCTGAGCGCCAGCCGC
TGCAGCGAGAAGGCCCAGGCCTTCGCCGAGCGCCTGGACCCCGTGCGCCTGAACGGCAGCACCGTGGAGTTCAGCAGCGAGTTCTTCACCGGCAACAACGAGCAGCAGC
TGCGCCTGCTGTACGAGAACGGCGAGAGCGTGCTGACCTTCCGCGACGGCGCCCGCGGCGCCCACCCCGACGAGACCTTCAGCAAGGGCATCGTGAACCCCCCCGAGAG
CTTCGAGGTGGCCGTGGTGCTGCCCGAGCAGCAGGCCGACACCTGCAAGGCCCAGTGGGACACCATGGCCGACCTGCTGAACCAGGCCGGCGCCCCCCCCACCCGCAGC
GAGACCGTGCAGTACGACGCCTTCAGCAGCCCCGAGAGCATCAGCCTGAACGTGGCCGGCGCCATCGACCCCAGCGAGGTGGACGCCGCCTTCGTGGTGCTGCCCCCCGA
CCAGGAGGGCTTCGCCGACCTGGCCAGCCCCACCGAGACCTACGACGAGCTGAAGAAGGCCCTGGCCAACATGGGCATCTACAGCCAGATGGCCTACTTCGACCGCTTCC
GCGACGCCAAGATCTTCTACACCCGCAACGTGGCCCTGGGCCTGCTGGCCGCCGCCGGCGGCGTGGCCTTCACCACCGAGCACGCCATGCCCGGCGACGCCGACATGTTCA
TCGGCATCGACGTGAGCCGCAGCTACCCCGAGGACGGCGCCAGCGGCCAGATCAACATCGCCGCCACCGCCACCGCCGTGTACAAGGACGGCACCATCCTGGGCCACAGC
AGCACCCGCCCCCAGCTGGGCGAGAAGCTGCAGAGCACCGACGTGCGCGACATCATGAAGAACGCCATCCTGGGCTACCAGCAGGTGACCGGCGAGAGCCCCACCCACAT
CGTGATCCACCGCGACGGCTTCATGAACGAGGACCTGGACCCCGCCACCGAGTTCCTGAACGAGCAGGGCGTGGAGTACGACATCGTGGAGATCCGCAAGCAGCCCCAGA
CCCGCCTGCTGGCCGTGAGCGACGTGCAGTACGACACCCCCGTGAAGAGCATCGCCGCCATCAACCAGAACGAGCCCCGCGCCACCGTGGCCACCTTCGGCGCCCCCGAG
TACCTGGCCACCCGCGACGGCGGCGGCCTGCCCCGCCCCATCCAGATCGAGCGCGTGGCCGGCGAGACCGACATCGAGACCCTGACCCGCCAGGTGTACCTGCTGAGCCA
GAGCCACATCCAGGTGCACAACAGCACCGCCCGCCTGCCCATCACCACCGCCTACGCCGACCAGGCCAGCACCCACGCCACCAAGGGCTACCTGGTGCAGACCGGCGCCTT
CGAGAGCAACGTGGGCTTCCTG
```

Figure S11. Plasmid map and NgAgo CDS of the pNgAgo-EGFP construct.

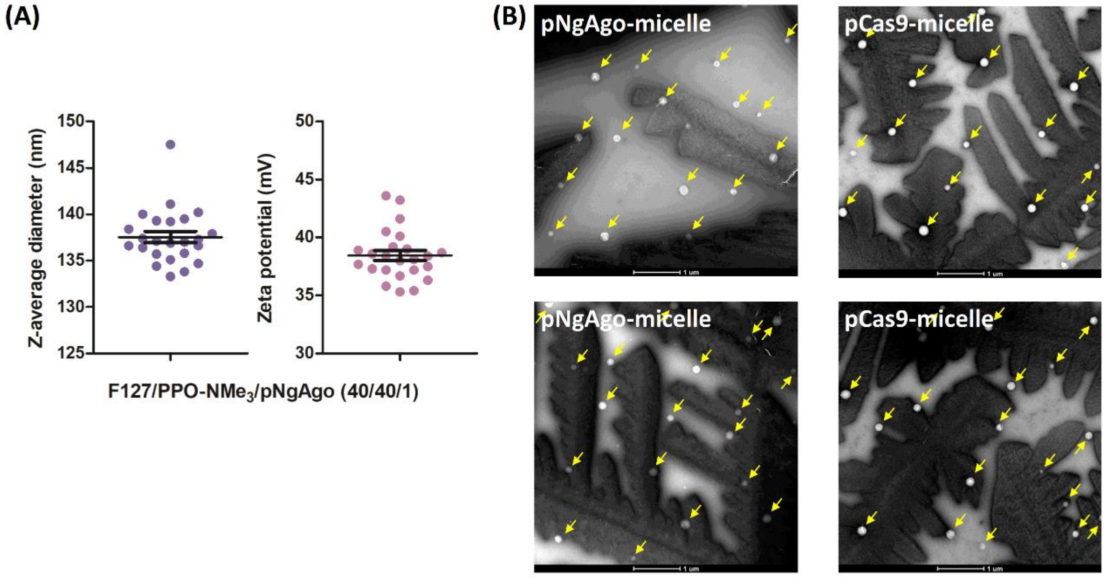

Figure S12. Physicochemical characterization of pNgAgo-EGFP-encapsulated micelle. (A) Size and zeta potential of F127/PPO-NMe$_3$/pNgAgo micelle (40/40/1). Data are presented as average ± SEM (n = 24). (B) Representative TEM images of pNgAgo- and pCas9- encapsulated micelles. Micelles were marked in each image. Scale bar represents 1 μm.

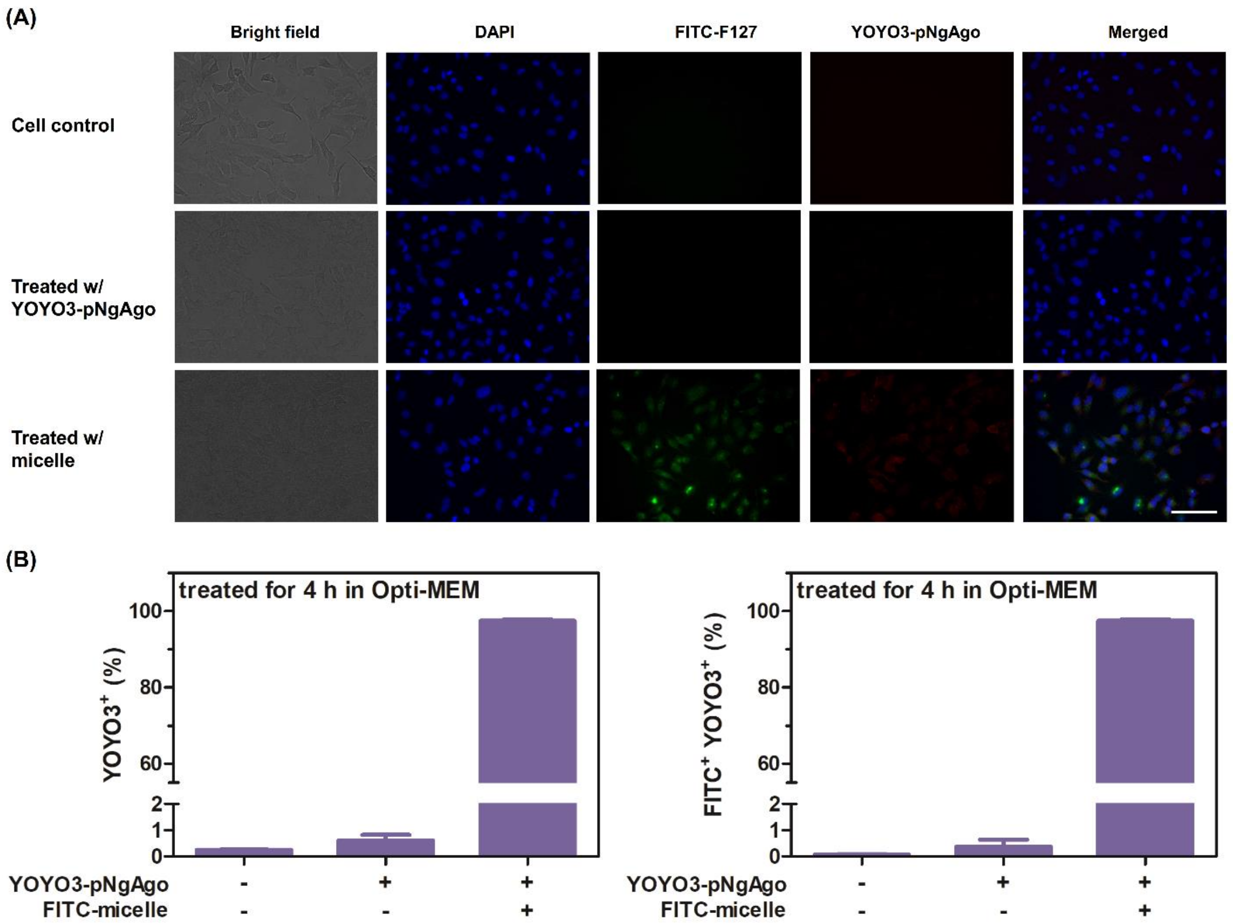

Figure S13. Cellular uptake of pNgAgo-encapsulated micelle. (A) Fluorescent images of the HeLa cells treated with FITC-F127/PPO-NMe_3/YOYO3-pNgAgo micelle for 4 h. Scale bar represents 100 μm. (B) Total plasmid-accessible cell % and (C) micelle-mediated plasmid-accessible cell %, quantified using FACS. For the cellular uptake study, cells were seeded in a 24-well plate one day prior to transfection. The NgAgo plasmid (Addgene #78253) was first stained with YOYO3 and complexed with PPO-NMe_3 and FITC-F127. Cells were treated with the micelle for 4 h in Opti-MEM and then either fixed for microscope measurement or harvested for FACS analysis. Cellular uptake rate (YOYO3$^+$% and FITC$^+$ YOYO3$^+$ %) are presented as average ± SEM (*n=3*).

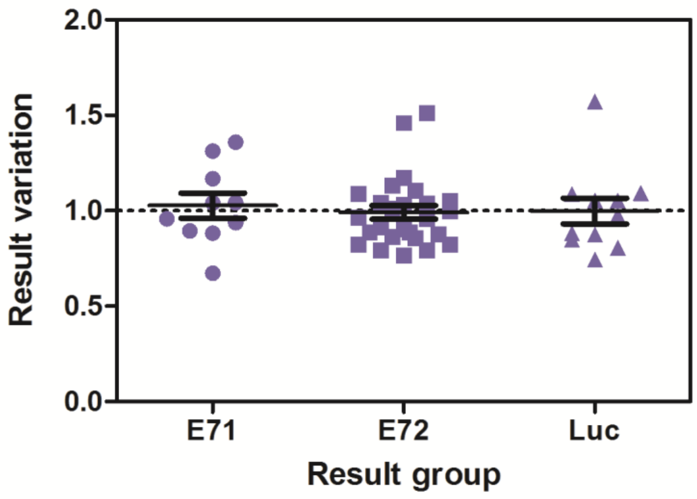

Figure S14. Validation on real-time PCR result variation. The result variation was defined as the ratio of the mRNA expression level determined using the previously reported primers (qPCR_E7_F2/R2) to that using the primers used in this study (qPCR_E7_F1/R1). For each group, the variation was calculated from at least 10 individual results. Data are presented as average ± SEM.

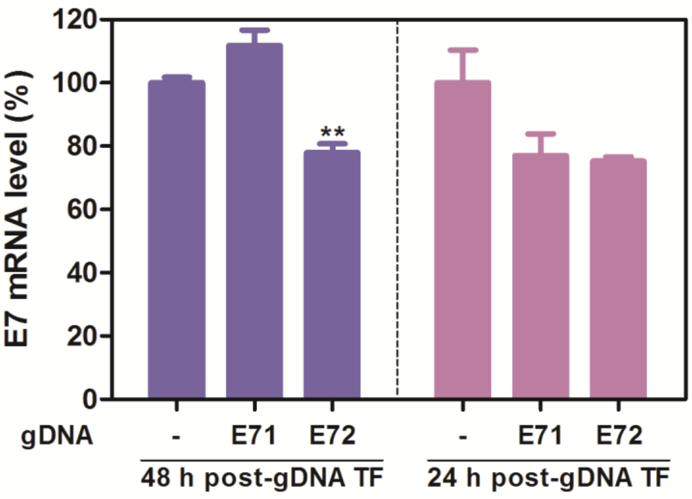

Figure S15. HPV18-E7 gene knockdown with Lipofectamine-delivered NgAgo. NgAgo and corresponding gDNAs were delivered using Lipofectamine 2000. Transfection and gene knockdown validation were carried out with the protocol mentioned in the Experimental section. Data are presented as average ± SEM (n = 3). One-way ANOVA with Dunnett's multiple comparison test was used for p-value calculation. The significant level is represented as ∗∗ ($p < 0.01$). (abbreviation used in the figure: TF, transfection)

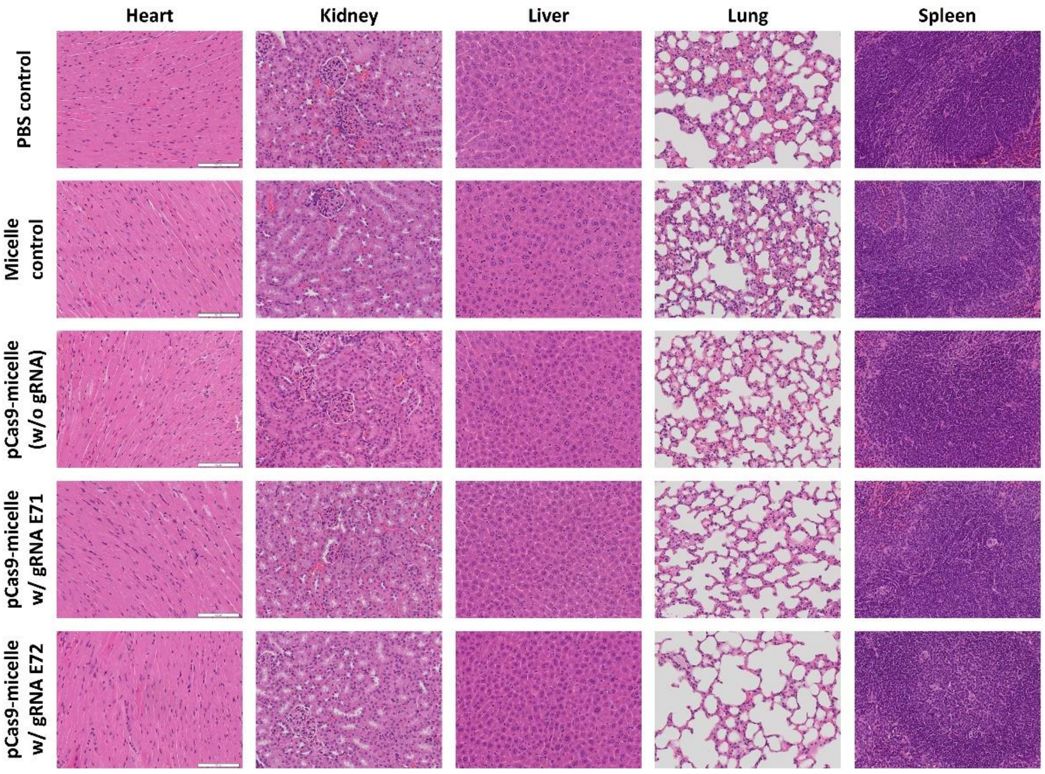

Figure S16. H&E staining for the major organs extracted from the pCas9-micelle-treated mice. Scale bar represents 100 μm.

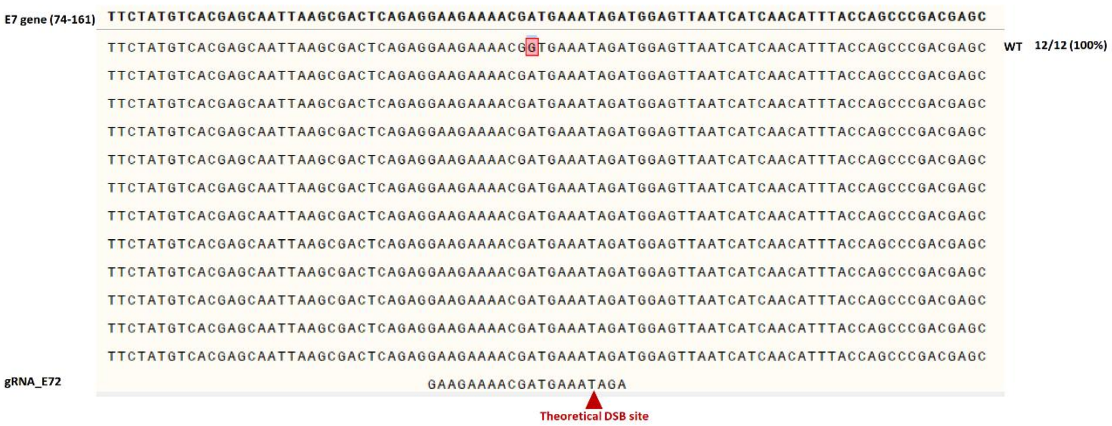

Figure S17. Sequencing validation of the extracted tumor from the Cas9 control group. Sequencing was done by Eton Bioscience with the primer pUC19_SEQ_F.

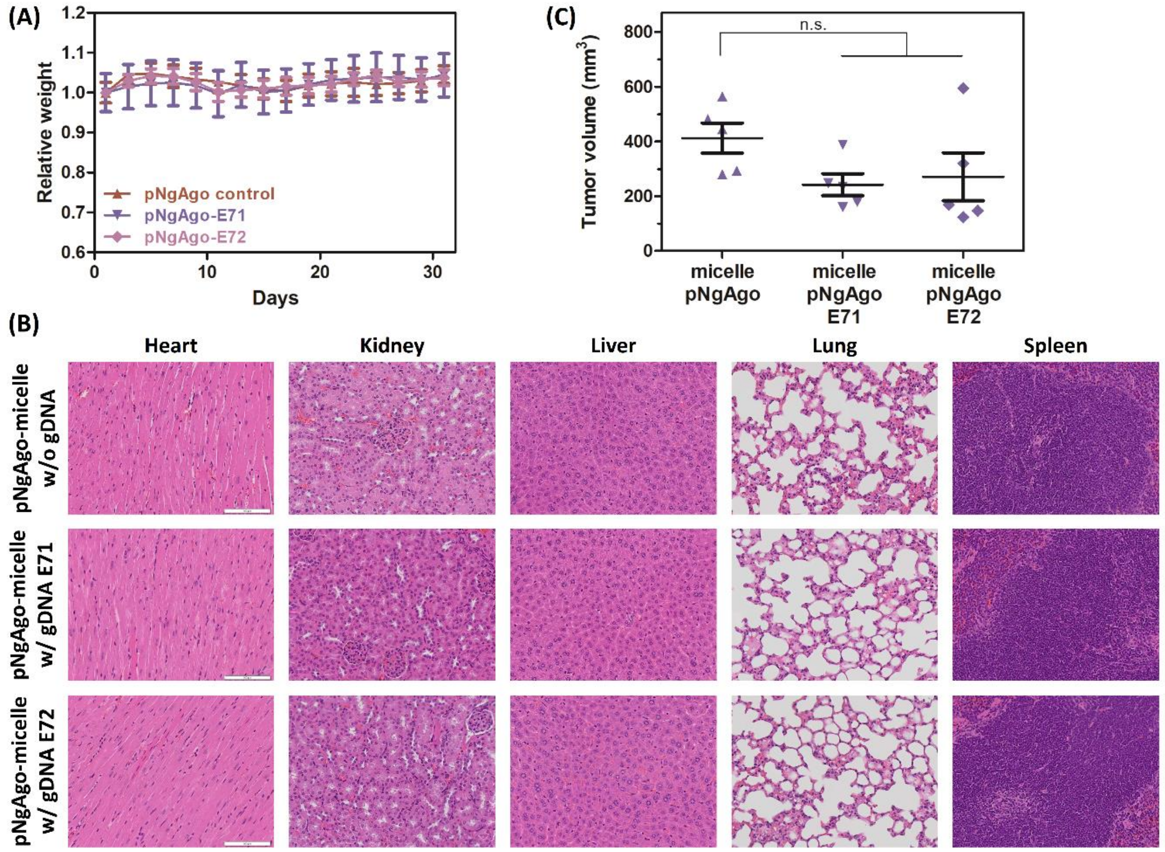

Figure S18. *In vivo* evaluation of micelle-delivered NgAgo. (A) Changes in body weight throughout the whole treatment course. (B) Tumor volume comparison on the day when mice were sacrificed (Day 31). Data are presented as average ± SEM (n = 5). One-way ANOVA with Dunnett's multiple comparison test was used for p-value calculation. The significant level is represented as n.s. (no significance). (C) Representative images of the H&E stained organs extracted from the pNgAgo-micelle-treated mice. Scale bar represents 100 μm.

Table S1. Sequences of the primers and gDNAs used in this study

Primer	Sequence	Purpose	Amplicon size
pUC19_E7F<br>pUC19_E7R	CGGTACCCGGGGATCGGTGCCAGAAACCGTTGAAT<br>CGACTCTAGAGGATCCCTCCCCGTCTGTACCTTCT	HPV E7 Gene disruption detection	551 bp
pUC19_OF_E71F<br>pUC19_OF_E71R	CGGTACCCGGGGATCTCTAAGAAGGCCGCATGAGT<br>CGACTCTAGAGGATCGAAAGATTGCTCACCCTCCTG	Off-target detection (for gRNA E71; on chromosome 3; predicted by Cas-OFFinder)	674 bp
pUC19_OF_E72F<br>pUC19_OF_E72R	CGGTACCCGGGGATCGTGTCTTCTCATTTCATCCTGCAAA<br>CGACTCTAGAGGATCTGGGGATCAAGATGGCCTTAC	Off-target detection (for gRNA E72; on chromosome 5; predicted by Cas-OFFinder)	636 bp
pUC19_OTA_ch2F<br>pUC19_OTA_ch2R	CGGTACCCGGGGATCGCCCACAGCTGACAAACATTTA<br>CGACTCTAGAGGATCTCGGATAAGGAAAGATGACCAGA	Off-target detection (for gRNA E72; on chromosome 2; predicted using BLAST)	823 bp
pUC19_OTA_ch6F<br>pUC19_OTA_ch6R	CGGTACCCGGGGATCACCACTGGTCTAGGTCTCAAG<br>CGACTCTAGAGGATCACCCTTGGGCTATAGTAGTTGGA	Off-target detection (for gRNA E72; on chromosome 6; predicted using BLAST)	905 bp
pUC19_OTA_ch10F<br>pUC19_OTA_ch10R	CGGTACCCGGGGATCCCCGGACAAACACCAGATGAAT<br>CGACTCTAGAGGATCGAGTGTATTGCCCTGACTCATT	Off-target detection (for gRNA E72; on chromosome 10; predicted using BLAST)	819 bp
pUC19_SEQ_F	ACGTTGTAAAACGACGGCCAGTGA	Sequencing primer for identifying the Cas9-induced mutations	
LKO.1 5’	GACTATCATATGCTTACCGT	Sequencing primer for verifying pCas9 constructs	
gDNA E71	CTGAGTCGCTTAATTGCTCGTG	HPV18-E7 targeting gDNA	
gDNA E72	ATCTATTTCATCGTTTTCTTCC	HPV18-E7 targeting gDNA	
gDNA Luc	AACAACTTTACCGACCGCGCCC	Negative control gDNA (Luciferase-targeting)	
gDNA GFP	TGAAGAAGATGGTGCGCTCCTG	Negative control gDNA (EGFP-targeting)	
qPCR_E7_F1<br>qPCR_E7_R1	ATGAAATTCCGGTTGACCTTCT<br>CTCGTCGGGCTGGTAAATGTT	qPCR primers for HPV18-E7 detection	
qPCR_E7_F2<br>qPCR_E7_R2	CACGAGCAATTAAGCGACTCAGAG<br>ATGCACACCACGGACACACAAAGG	qPCR primers for HPV18-E7 detection	
qPCR_GADPH_F<br>qPCR_GADPH_R	GGAAGGTGAAGGTCGGAGTCA<br>GTCATTGATGGCAACAATATCCACT	qPCR primers for GADPH internal control	

* T7EI/sequencing primers were designed using NCBI Primer BLAST. All the gDNAs were 5’-phosphorylated. Adapter sequence on primer for cloning is highlighted in red.
